# Cell- and Fluid-Sampling Microneedle Patches for Monitoring Tissue-Resident Immunity

**DOI:** 10.1126/scitranslmed.aar2227

**Published:** 2018-11-14

**Authors:** Anasuya Mandal, Archana V. Boopathy, Lionel K.W. Lam, Kelly D. Moynihan, Mary E. Welch, Nitasha R. Bennett, Michelle E. Turvey, Nikki Thai, Jenny H. Van, J. Christopher Love, Paula T. Hammond, Darrell J. Irvine

**Affiliations:** 1 Department of Chemical Engineering, MIT, Cambridge, MA 02139, USA.; 2 Koch Institute for Integrative Cancer Research, MIT Cambridge, MA 02139, USA.; 3 Department of Biological Engineering, MIT, Cambridge, MA 02139, USA.; 4 Infectious Diseases IRG, Singapore-MIT Alliance for Research and Technology, Singapore 138602.; 5 Department of Materials Science and Engineering, MIT, Cambridge, MA 02139, USA.; 6 Institute for Soldier Nanotechnologies, MIT, Cambridge, MA 02139, USA.; 7 Ragon Institute of MGH, MIT, and Harvard, Cambridge, MA 02139, USA.; 8 Howard Hughes Medical Institute, MD 20815, USA.

## Abstract

Important immune cell populations reside within tissues and are not accessed by traditional blood draws used to monitor the immune system. To address this issue at an important barrier tissue, the skin, we created a microneedle-based technology for longitudinal sampling of cells and interstitial fluid, enabling minimally-invasive parallel monitoring of cellular and humoral immune responses. Solid microneedle projections were coated by a crosslinked biocompatible polymer, which swells upon skin insertion, forming a porous matrix for local leukocyte infiltration. By embedding molecular adjuvants and specific antigens encapsulated in nanocapsules within the hydrogel coating, antigen-specific lymphocytes can be enriched in the recovered cell population, allowing for subsequent detailed phenotypic and functional analysis. We demonstrate this approach in two animal models: mice immunized with a model protein antigen or infected in the skin with vaccinia virus. Following vaccination or infection, sampling microneedles allowed tissue-resident memory T (T_RM_) cells to be longitudinally monitored in the skin for many months, during which time the antigen-specific T cell population in systemic circulation contracted to low or undetectable counts. Importantly, sampling microneedles did not change the immune status of naïve or antigen-exposed animals. We also validated the ability of cell sampling using human skin samples. This approach may be useful in vaccines and immunotherapies to temporally query T_RM_ populations, or as a diagnostic platform to sample for biomarkers in chronic inflammatory and autoimmune disorders, allowing information previously accessible only via invasive biopsies to be obtained in a minimally-invasive manner from the skin or other mucosal tissues.

## One Sentence Summary

A minimally-invasive microneedle device allows repeated sampling of tissue-resident immune cell populations from the skin.

## Introduction

Current methods for accessing compartments of the body to obtain samples of tissues, cells, or fluid for medical diagnosis and monitoring fall into three categories: (i) invasive (such as via traditional phlebotomy), (ii) minimally-invasive (such as saliva swabs) and, (iii) non-invasive (such as urine collection). Immune monitoring is performed primarily by analysis of blood draws, a practice in use since the early times (1), with analysis by flow cytometry of peripherally-sampled blood as the most prevalent method for immunophenotyping (2, 3). However, many important immune cell populations preferentially reside in peripheral tissues including barrier tissues such as the skin, gut, and other mucosal surfaces, and do not recirculate in the blood. This includes tissue-resident macrophages, natural killer cells, NK T cells, B cells, plasma cells, and memory T cells (4-6). Notably, a larger proportion of T cells reside in peripheral tissues than in the secondary lymphoid organs at steady state (7). Resident memory T cells (T_RM_s) reside in the skin and other mucosal tissues without recirculation in the blood (8), and include cytotoxic CD8^+^ T cells poised for immediate interception and killing of infected cells, providing a critical frontline response to infection (9). These cells are not accessed by traditional blood-based analysis of the immune response.

Current approaches for monitoring tissue-resident immune cell populations are limited. One widely used method to query the skin is via delayed type hypersensitivity tests such as the Mantoux test (10) and allergen patch tests (11, 12), which offer qualitative readouts of an immune response towards a particular antigen. In the case of the tuberculin Mantoux test, the tuberculin antigen is injected into the dermis and the skin is monitored for the next 2-3 days for the development of an induration, which indicates the presence of a recall immune response. However, these methods fail to offer quantitative information about phenotypic and functional aspects of the cell infiltrate. There exist invasive methods of sampling immune cell populations from lymph nodes (13) or from skin (14) but these are apparatus-intensive and require special training for use. Minimally-invasive methods for quantitative monitoring of the immune status of the skin or other barrier tissues could provide valuable information in the context of patients with genetic and acquired immunodeficiency disorders, organ transplants, and vaccination, but such approaches are currently lacking.

Microneedle patches, arrays of typically sub-mm pyramidal or conical projections designed to mechanically pierce the stratum corneum and reach the viable epidermis/upper dermis, have been extensively explored for delivery of drugs and vaccines into the skin (15). However, a variety of microneedles have also been designed to extract interstitial fluid (ISF) from the skin, enabling monitoring of glucose, antibodies, or other biomarkers, including solid microneedles to simply perforate the skin and enable access to ISF (16). While these are effective minimally-invasive technologies, to date only sampling of soluble biomolecules has been demonstrated.

Here we demonstrate a microneedle-based platform for the combined sampling of ISF and viable cells from the skin. Using solid microneedles coated by a sampling hydrogel layer that swells on application to the skin, we demonstrate recovery of leukocytes that infiltrate the sampling layer. Mimicking the classical Mantoux test, we further show that by incorporation of antigen- and adjuvant-carrying nanoparticles into the sampling layer, forming Stimulatory Sampling Microneedles (SSMNs), we can specifically enrich for antigen-specific T_RM_s and memory cells, without perturbing the immune status of animals. This technology thus provides an innovative means for minimally-invasive monitoring of key immune cell populations of interest in vaccination, infectious disease, and autoimmune disorders.

## Results

### Alginate-coated microneedles can be fabricated to sample cells and ISF

**Figure 1:**
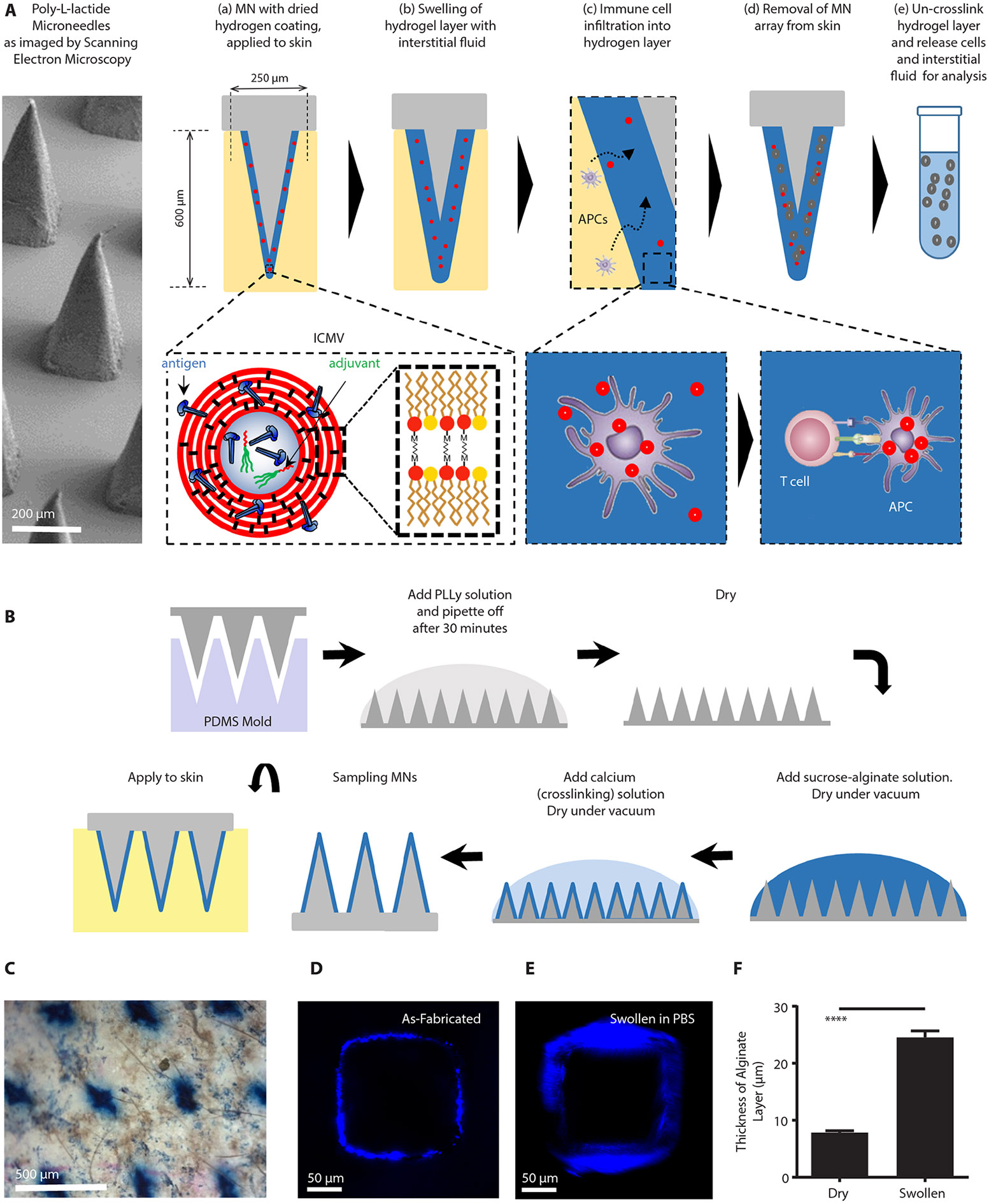
Fabrication of Stimulatory Sampling Microneedles (SSMNs) platform for immune monitoring. **(A)** Schematic of SSMNs structure and proposed mechanisms of action. **(B)** Microneedle fabrication process. **(C)** Trypan blue stain of mouse ear tissue. Scale bar 500 μm. (D-F) Confocal micrographs showing a cross section of the alginate layer (blue) on an individual microneedle projection before **(D)** and after **(E)** swelling in PBS for 20 minutes at 25°C (scale bar 50 μm). **(F)** Thickness of alginate layer quantified before/after PBS swelling.

The conceptual design we pursued is outlined in Fig. 1A: A skin patch comprised of a square array of pyramidal solid polymer microneedles (each 250 μm in width at the base and ~600 μm in height) is coated with a biocompatible hydrogel layer. Embedded within the gel layer are adjuvants and lipid nanocapsules containing an antigen of interest (Fig. 1A(a)). Upon application to the skin, the hydrogel layer swells with the intake of ISF (Fig. 1A(b)). Antigen presenting cells (APCs) migrate into the alginate matrix in response to the localized inflammation induced by microneedle penetration into the skin (17), are activated by the adjuvant and take up the embedded antigen-carrying nanocapsules (Fig. 1A(c)). Cytokines and chemokines produced by these activated APCs in turn promote recruitment of T cells into the gel coating. T cells specific for antigen presented by the nanocapsule-loaded APCs will be retained in the gel layer, enriching these antigen-specific cells. Upon removal of the microneedles from the skin, the hydrogel layer is dissolved, releasing cells for analysis by diverse immunological tools for phenotyping and immune profiling (Fig. 1A(d-e)). We hypothesized that such a design would simultaneously allow ISF to be sampled and provide a window into the antigen-specific immune cell populations in the skin.

To implement this design, polymer microneedle arrays were formed by melt-molding polylactide (18), and alginate was selected as a biocompatible hydrogel coating material (19). To select an alginate composition, we compared cellular infiltration into subcutaneously implanted alginate gels composed of low or high molecular weight (“Lo MW”, 75 KDa; and “Hi MW”, 200,000 KDa) and with different alginate concentrations. Basal cellular infiltration into alginate matrices was similar in Lo MW and Hi MW gels composed of 1 wt% alginate, which had an elastic modulus of ~1 KPa ensuring sufficient mechanical stiffness (Fig. S1A-B). Hi MW alginate was chosen for better mechanical integrity and ease of handling. Microneedles were first coated with an absorbed layer of polylysine to promote electrostatic adhesion of the alginate to the underlying microneedle surface, followed by dropcasting of an alginate/sucrose solution, crosslinking of the alginate coating by application of calcium chloride, and drying to a solid alginate/sucrose layer (Fig. 1B). Interbilayer-crosslinked multilamellar vesicles (ICMVs) (20), ~125 nm diam. lipid nanocapsules carrying antigen and adjuvant (described further below), were added to the alginate solution prior to dropcasting to incorporate these components into the alginate matrix. Sucrose was included in the gel layer to increase the mechanical integrity of the alginate coating during initial penetration of the stratum corneum and act as an *in situ* porogen, increasing the porosity of the alginate as it swells in the skin. Trypan blue staining confirmed that alginate/sucrose-coated microneedles efficiently penetrated the skin of mice (Fig. 1C). Microneedles exposed to PBS for 20 minutes showed rapid rehydration and swelling of the alginate layer by ~3-fold in thickness from its dried state (Fig. 1D-F). Additionally, we evaluated the potential for ISF sampling with the gel layer. *In vitro*, microneedles exposed to solutions of IgG or IgM and then digested for analysis of protein content by ELISA gave accurate measurements of the bulk solution concentration of IgG or IgM solutions (Fig. S2).

### Sampling microneedles can be utilized for the recovery of cells and ISF

We were particularly interested in sampling of antigen-specific skin T_RM_s, and thus we first established a model immunization protocol to generate a defined population of skin-resident T_RM_. Mice were vaccinated twice subcutaneously at the base of the tail, with ovalbumin protein (OVA) and lipid-conjugated CpG, a TLR9 agonist. Four weeks later, cell suspensions from blood and digested ear tissue were stained with CFSE to distinguish cells from tissue digest debris, and OVA-specific CD8^+^ T-cells expressing T_RM_ markers were identified by flow cytometry (Fig. S3A). We defined these cells conservatively as CD69^+^CD103^+^ T cells, although it is known that T_RM_ may lack expression of one or both of these markers in some tissues (7). High numbers of OVA-specific CD8^+^ T-cells were detected in the blood at this time point (Fig. S3B-C), and ear tissue showed the presence of antigen-specific CD69^+^CD103^+^ T_RM_s (Fig. S3B-D).

**Figure 2:**
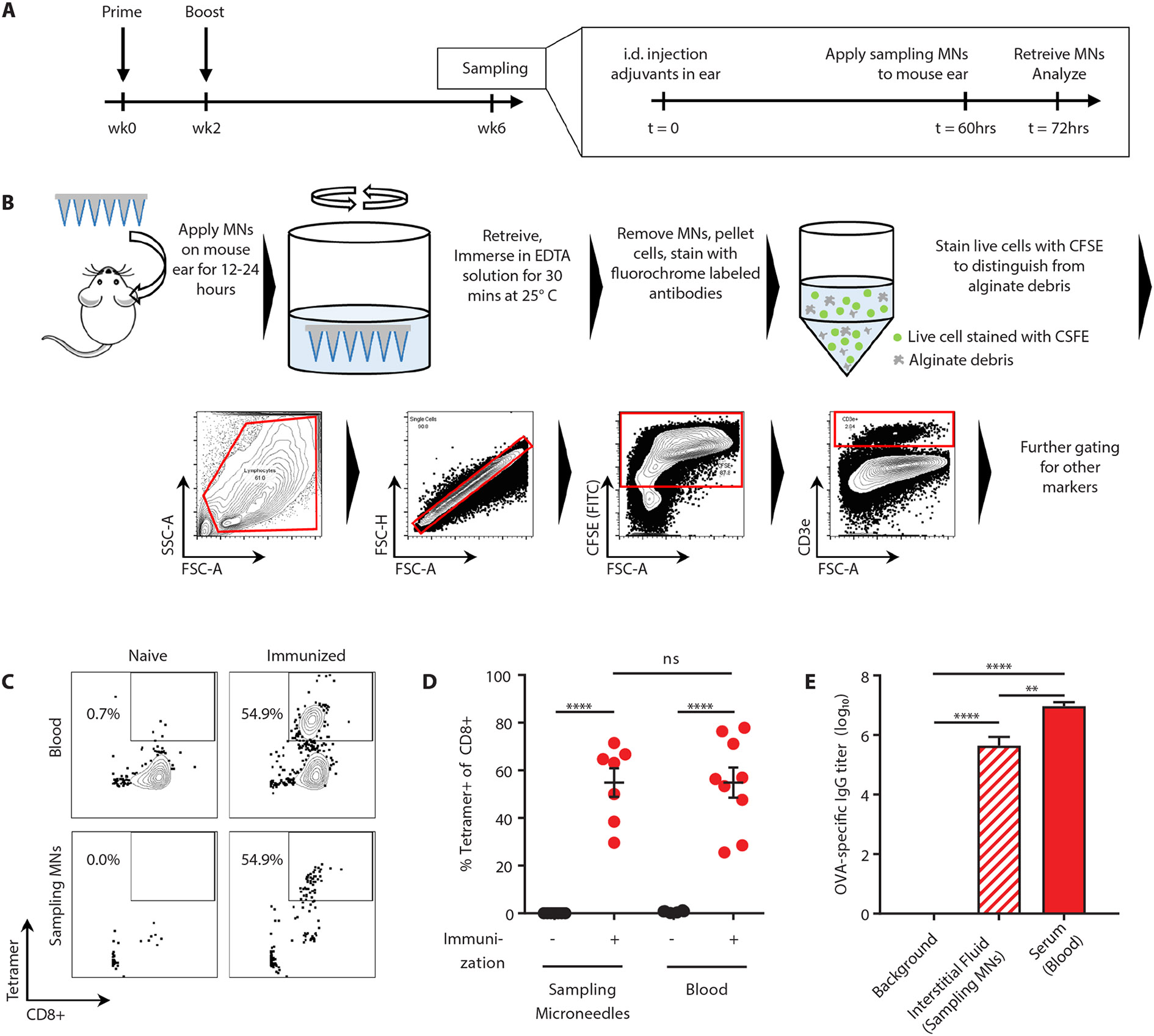
Cell sampling microneedles allow tandem analysis of cellular and humoral immune responses. **(A)** Groups of OVA-immunized or naïve C57Bl/6 mice (*n*=9/group) were injected intradermally in the ear at time zero with 2 μg OVA and 5 μg each of adjuvants polyI:C and pam3Cys. 60 hrs later, sampling microneedle were applied to the same site for 12 hrs, followed by retrieval and flow cytometry analysis. **(B)** Sample processing and flow cytometry gating strategy. **(C-D)** Representative flow cytometry plots (**C**) and quantification from groups of animals (**D**) showing OVA-specific SIINFEKL/H-2K^b^-streptavidin tetramer^+^ CD8^+^ cells, as sampled from blood or with cell-sampling microneedles. **(E)** OVA-specific IgG titers(log10) as quantified from serum or ISF from sampling microneedles. Data shown are means ± s.e.m. from one representative of two independent experiments. ns, nonsignificant, **, p < 0.01, ****, p < 0.0001, analyzed by one-way ANOVA, followed by Tukey’s HSD.

To evaluate the potential of alginate-coated microneedles to sample tissue-resident cells and ISF, we next modeled a classic delayed type hypersensitivity/Mantoux test (Fig. 2A). Naïve or OVA-immunized mice were injected intradermally in the ear with OVA and adjuvant. Five days later, alginate-coated microneedles containing no antigen or adjuvants in the gel coating were applied for 12 hours to the same skin site, then retrieved for analysis. To retrieve sampled cells, the alginate layer was dissolved in the presence of EDTA, recovered cells were stained with antibodies and labeled with CFSE to aid in distinguishing live cells from alginate debris, and analyzed by flow cytometry (Fig. 2B). As shown in Fig. 2C-D, naïve mice showed no detectable OVA-specific CD8^+^ T-cells in the blood, whereas OVA-specific T-cells were present at high numbers in immunized mice even at 4 weeks post-vaccination. Sampling microneedles also recovered a substantial tetramer^+^ population at the site of the antigen injection from immunized animals, though with lower total numbers of cells collected compared to the blood sample (Fig. 2C-D). We then compared OVA-specific IgG titers by ELISA on ISF recovered from the same microneedle patches vs. serum samples from the same animals. Consistent with prior studies comparing serum and ISF (21, 22), OVA-specific IgG was easily detected in microneedle-recovered ISF from immunized mice, at a lower titer than serum from the same animals (Fig. 2E).

### Adjuvant incorporation increases cell recovery by sampling microneedles

To simplify from the two-step sampling strategy performed in the experiments of Fig. 2, we next investigated whether a one-time application of microneedles loaded with molecular adjuvants would lead to effective cell recovery. Based on the expression of Toll-like receptors (TLRs) in keratinocytes and skin-derived dendritic cells, we tested the TLR3 agonist polyI:C and TLR1/2 agonist pam3Cys (23, 24). Intradermal injection of pam3Cys led to recruitment of CD8+ T cells and myeloid cells into the skin of C57Bl/6 mice, which was substantially augmented by co-administration of polyI:C (Fig. S4A-B). Inclusion of this adjuvant combination in alginate hydrogels implanted subcutaneously in eGFP mice (expressing GFP in all nucleated cells) also resulted in significantly higher (p < 0.05) cell infiltration into the gel compared to alginate alone (Fig. S4C-D).

PolyI:C is high molecular weight double-stranded RNA, which we expected to be retained effectively in the microneedle alginate coating, but pam3Cys is a small lipopeptide which may not be retained due to rapid diffusion from the gel layer. To promote retention of the latter and co-delivery of adjuvant signals with incorporated antigen to infiltrating APCs, we prepared pam3cys-loaded ICMV lipid capsules (mean diameter of 126 ± 11 nm, Fig. S5A). We then assessed cellular recruitment into the gel layer of microneedles carrying pam3Cys (either free or encapsulated in ICMVs) together with polyI:C in the alginate coating. As shown in Fig. S5B, incorporation of pam3Cys and polyI:C into the alginate coating increased cellular infiltration into the microneedle, and incorporation of pam3Cys into ICMVs further enhanced cell recruitment ~8-fold over microneedles carrying free pam3Cys. ICMV nanocapsules have a high degree of stability in physiological conditions, showing very low loss of encapsulated cargo over several days in the presence of serum (20). As shown in Fig. S6A, there was no significant drop in the quantity of polyI:C recovered from microneedles post 24 hr application to murine ear skin, and polyI:C recovered from microneedles stimulated TLR3-expressing reporter cells in culture similarly to polyI:C extracted from as-fabricated patches (Fig. S6B). Thus, the polyI:C adjuvant is stable over the duration of sampling *in vivo*.

### Inclusion of antigen in the sampling layer enhances recovery of antigen-specific lymphocytes by sampling microneedles

Incorporation of adjuvants in the gel layer should promote nonspecific recruitment of leukocytes, but such nonspecific cell sampling does not reflect the enrichment of antigen-specific lymphocytes that occurs rapidly at a site of infectious challenge (25). Thus, we next introduced specific antigens into ICMVs carried in the gel coating, co-encapsulating pam3Cys and the model antigen OVA to generate Stimulatory Sampling Microneedles (SSMNs). Dendritic cells can cross present exogenous protein antigen to CD8 T cells within a few hours of uptake (26). As illustrated in Fig. 1A, our expectation was that APCs taking up antigen in the alginate matrix would present peptides *in situ* to recruited antigen-specific T cells, arresting their migration and egress from the alginate matrix in response to TCR signaling and thereby enriching for antigen-specific cells in the sampled cell population.

**Figure 3:**
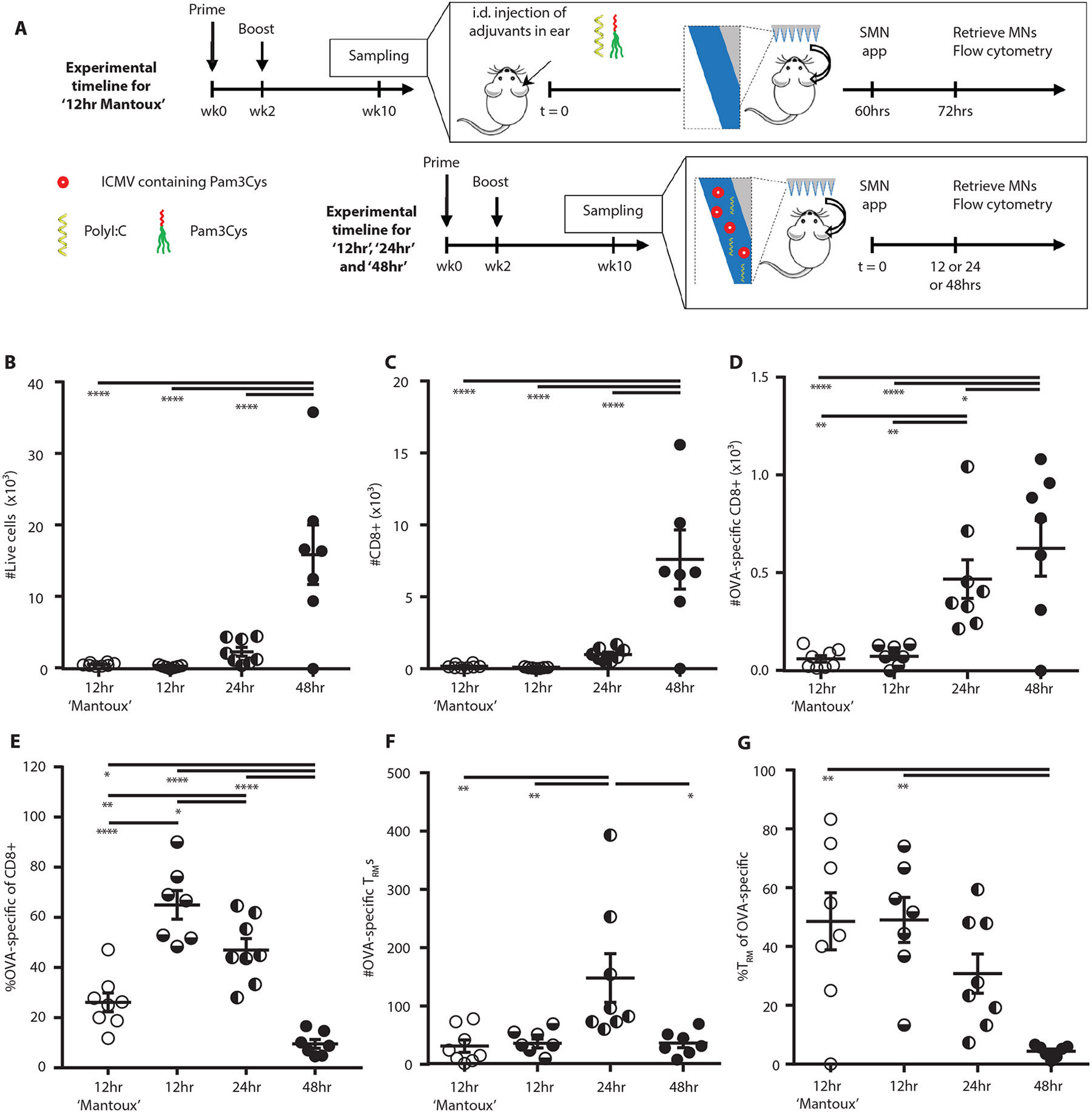
Microneedles incorporating adjuvants enable single-step sampling of antigen-specific T_RM_s. **(A)** Timeline of sampling optimization in C57Bl/6 mice (*n*=5/group). **(B-G):** Enumeration of recovered total live cells **(B)**, CD8^+^ cells **(C)**, OVA-specific CD8^+^ cells **(D)**, frequency of OVA-specific cells **(E),** OVA-specific T_RM_s **(F)**, and frequency of T_RM_s among OVA-specific cells **(G)**. Cell counts are per microneedle patch. Data shown are mean ± s.e.m. from one representative of three independent experiments. *, p<0.05, **, p<0.01, and ****, p < 0.0001 analyzed by one-way ANOVA, followed by Tukey’s HSD.

To test this idea, SSMNs were prepared with alginate coatings carrying polyI:C and ICMVs co-loaded with pam3Cys and OVA. SSMNs were applied for 12, 24 or 48 hours on the ears of OVA-immunized mice. These SSMN sampling groups were compared to animals sampled by a Mantoux-type experiment, where antigen and adjuvant were injected i.d. 60 hrs prior to application of microneedles carrying no stimuli in the alginate coating (SMNs, Fig. 3A). As shown in Fig. 3B-C, 12 hr application of SSMNs to the skin of mice receiving no pre-treatment recovered total live cells and CD8^+^ T-cells in numbers comparable to alginate-only SMNs applied in the two-step “Mantoux” setting. Application of SSMNs for 24 hr increased the cell recovery another ~3-fold whereas sampling for 48 hrs increased cell recovery ~8 fold (Fig. 3B-C). On average, ~9-10x more OVA-specific T cells were retrieved by SSMNs applied for 24 hr compared to 12 hrs, with only a minor further gain in OVA-specific cells for a 48 hr application time (Fig. 3D-E). Importantly, microneedles applied for 24 hrs maximized the recovery of CD3^+^CD8^+^CD69^+^CD103^+^OVA-specific T_RM_ cells (Fig. 3F-G). Among OVA-specific T cells, we noted that a longer SSMN application time resulted in the recovery of more T cells recruited from the systemic circulation, and a decreasing proportion of OVA-specific T_RM_s (Fig. 3G). Based on these findings we chose to focus on 24 hr SSMN sampling times, to maximize T_RM_ sampling.

**Figure 4:**
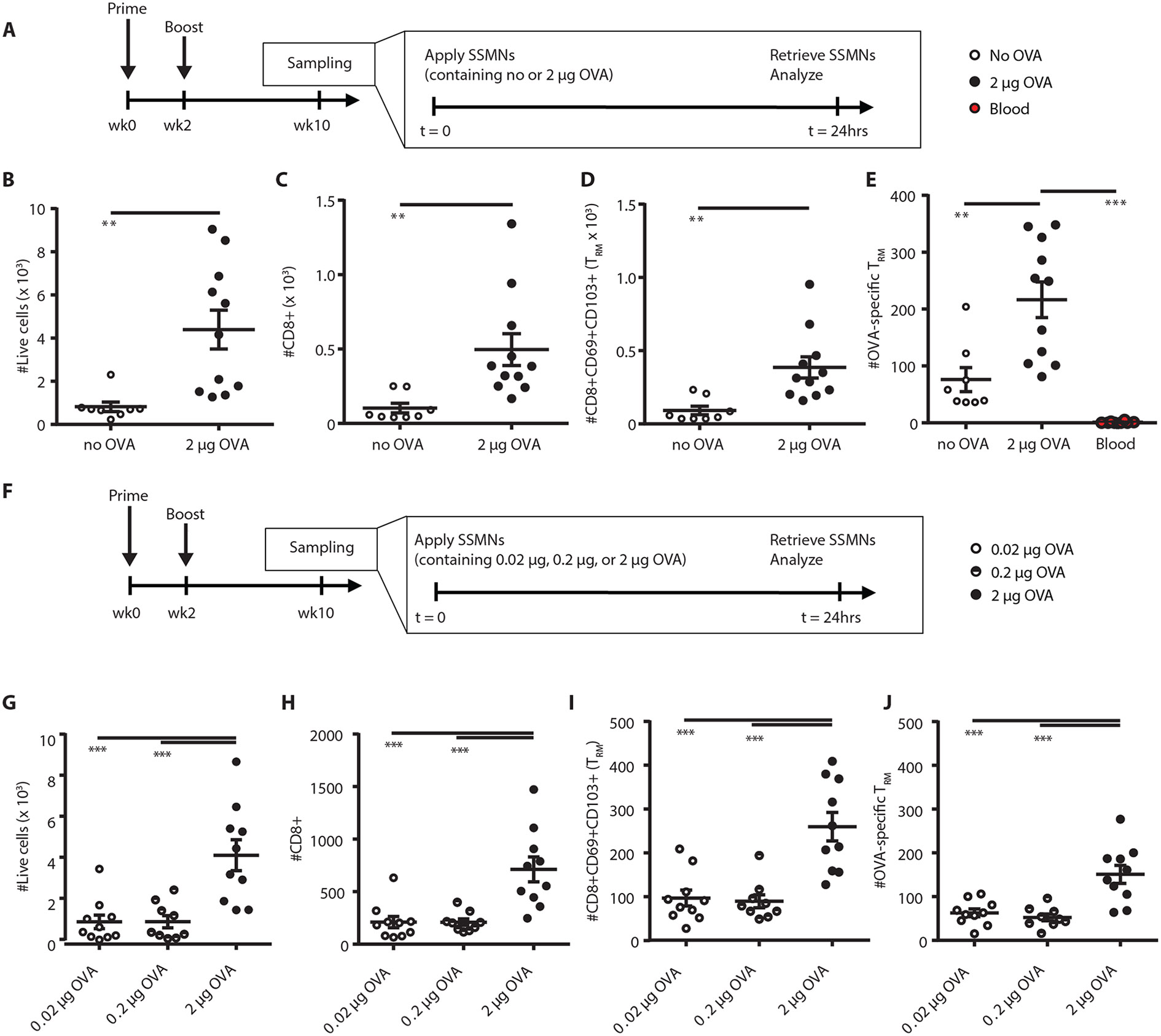
SSMNs containing antigen-loaded nanocapsules enrich sampling for antigen-specific T_RM_s. **(A-E)** Groups of OVA-immunized C57Bl/6 mice (*n*=6/group) were sampled with SSMN (Stimulatory Sampling Microneedle) arrays applied to the ear according to the timeline **(A)**. Enumeration of recovered total live cells **(B)**, CD8^+^ cells **(C)**, CD8^+^CD69^+^CD103^+^ T_RM_s **(D)** and OVA-specific T_RM_s **(E)**, per SSMN array, from SSMNs containing empty ICMV nanocapsules or ICMVs loaded with 2 μg OVA. **(F-J)** Groups of OVA-immunized C57Bl/6 mice (*n*=5/group) were sampled with microneedles applied to the ear according to the timeline **(F)**. Enumeration of recovered total live cells **(G)**, CD8^+^ cells **(H)**, CD8^+^CD69^+^CD103^+^ T_RM_s **(I)** and OVA-specific T_RM_s **(J)**, per SSMN array, from SSMNs containing ICMVs encapsulating 0.02 μg, 0.2 μg or 2 μg OVA. Data shown are mean ± s.e.m. from one representative of 2-3 independent experiments. **, p<0.01, *** and p < 0.001 analyzed by one-way ANOVA, followed by Tukey’s HSD.

We next evaluated how antigen inclusion in the cell-sampling layer affected the composition of recovered leukocytes. As expected, antigen-loaded microneedles were more effective in recovering antigen-specific T-cells, but also led to a generally higher recovery of total leukocytes (Fig. 4A-E). This may reflect rapid activation of antigen-specific T_RM_s recruited to the microneedles, which would both arrest these cells in the gel layer and trigger production of additional cytokines/chemokines (27). We next titrated the dose of OVA incorporated in the microneedles, holding the adjuvant dose constant (Fig. 4F). A notable increase in recovered antigen-specific and non-specific T cells was observed when the microneedles carried 2 μg of OVA compared to the lower doses of antigen (Fig. 4G-J). Approximately 5,000 live cells were typically recovered from a single microneedle array loaded with 2 μg antigen. Based on these studies, a 24 hour application with cell-sampling microneedles incorporating 2 μg of antigen in ICMVs in the crosslinking layer of the alginate coating was designated as the optimized sampling strategy.

**Figure 5:**
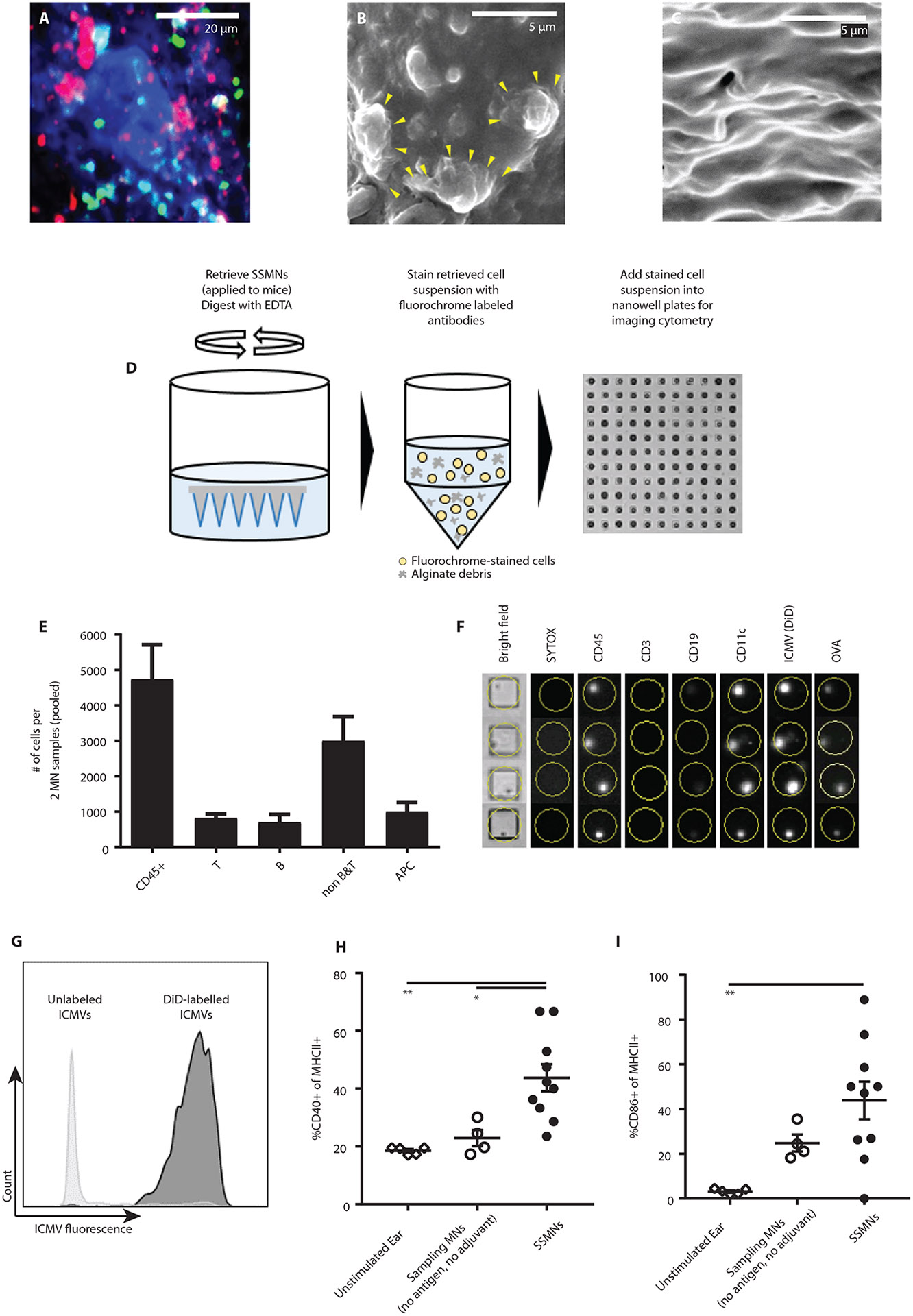
Sampling microneedles containing ICMV nanocapsules accumulate antigen-loaded dendritic cells. **(A)** Confocal micrograph of SSMN array surface showing alginate (blue), embedded ICMVs (red), and recruited cells (green), following application to the skin of eGFP mice. Scale bar 20 μm. **(B)** SEM of SSMNs post-application to mouse skin for 24 hours showing lymphocytes, approximately 5μm in diameter, **(C)**. **(D-G)** SSMNs containing 5 μg of polyI:C and DiD-labeled ICMVs encapsulating 2 μg Alexa Fluor555-labeled OVA and 5 μg pam3Cys were applied to the ears of OVA-immunized mice (*n*=3/group) for 24 hours, followed by retrieval, antibody staining and phenotypic analysis via imaging cytometry **(D). (E)** Cell phenotypes obtained from SSMNs. **(F)** Representative imaging cytometry data showing overlay of fluorescent channels for live/dead dye Sytox, CD45, CD3, CD19, CD11c, DiD (ICMV) and OVA-Alexa Fluor555. Dimension of each well is 50 μm. **(G)** Representative 2D imaging cytometry plots showing OVA/ICMV fluorescence in CD45^+^CD11c^+^ dendritic cells. **(H-I)** Expression of activation markers CD40 (**H**) and CD86 (**I**) among APCs recovered by microneedles. Data shown are mean ± s.e.m. from one representative of 2-3 independent experiments. *, p<0.05, **, and p<0.01 analyzed by one-way ANOVA, followed by Tukey’s HSD.

Confocal microscopy of SSMNs following skin sampling of OVA-immunized eGFP mice showed the presence of cells infiltrating the alginate matrix in close proximity to aggregates of ICMV particles (Fig. 5A). Scanning electron microscopy also revealed lymphoid cells displaying lamellar protrusions embedded within the matrix, which were absent in control samples containing neither antigen nor adjuvant (Fig. 5B-C). To determine whether infiltrating APCs interacted with ICMVs embedded within the alginate layer, we employed an image-based cytometry methodology: Cells recovered by SSMNs were seeded in a nanowell array, stained with antibodies, and subsequently imaged by high throughput microscopy (Fig. 5D). Imaging cytometry analysis showed that 20% of cells recovered from OVA-immunized mice were T-cells (CD3e^+^), 15% were CD19^+^ B cells, ~20% of recovered cells were CD45^+^CD3e^−^CD19^−^CD11c^+^, likely APCs, with the remaining cells likely myeloid cells and granulocytes (Fig. 5E). Micrographs of individual nanowells containing single cells showed live APCs which were positive for both ICMVs (DiD) and fluorescent OVA signals, suggesting uptake of OVA-ICMVs by recruited dendritic cells in the gel layer (Fig. 5F). Essentially all CD11c^+^ APCs recovered from the microneedles were ICMV^+^ (Fig. 5G). In addition, among MHCII^+^ antigen presenting cells recovered from SSMNs, expression of the activation markers CD40 and CD86 were considerably increased compared to cells recovered from unstimulated ear skin or cells sampled with microneedles containing no antigen/adjuvants (Figs. 5H-I, and Fig. S7). Thus, relevant APCs efficiently acquired the antigen- and adjuvant-loaded nanocapsules and become activated on infiltration into the sampling microneedle matrix.

### SSMNs do not immunize animals during sampling

**Figure 6:**
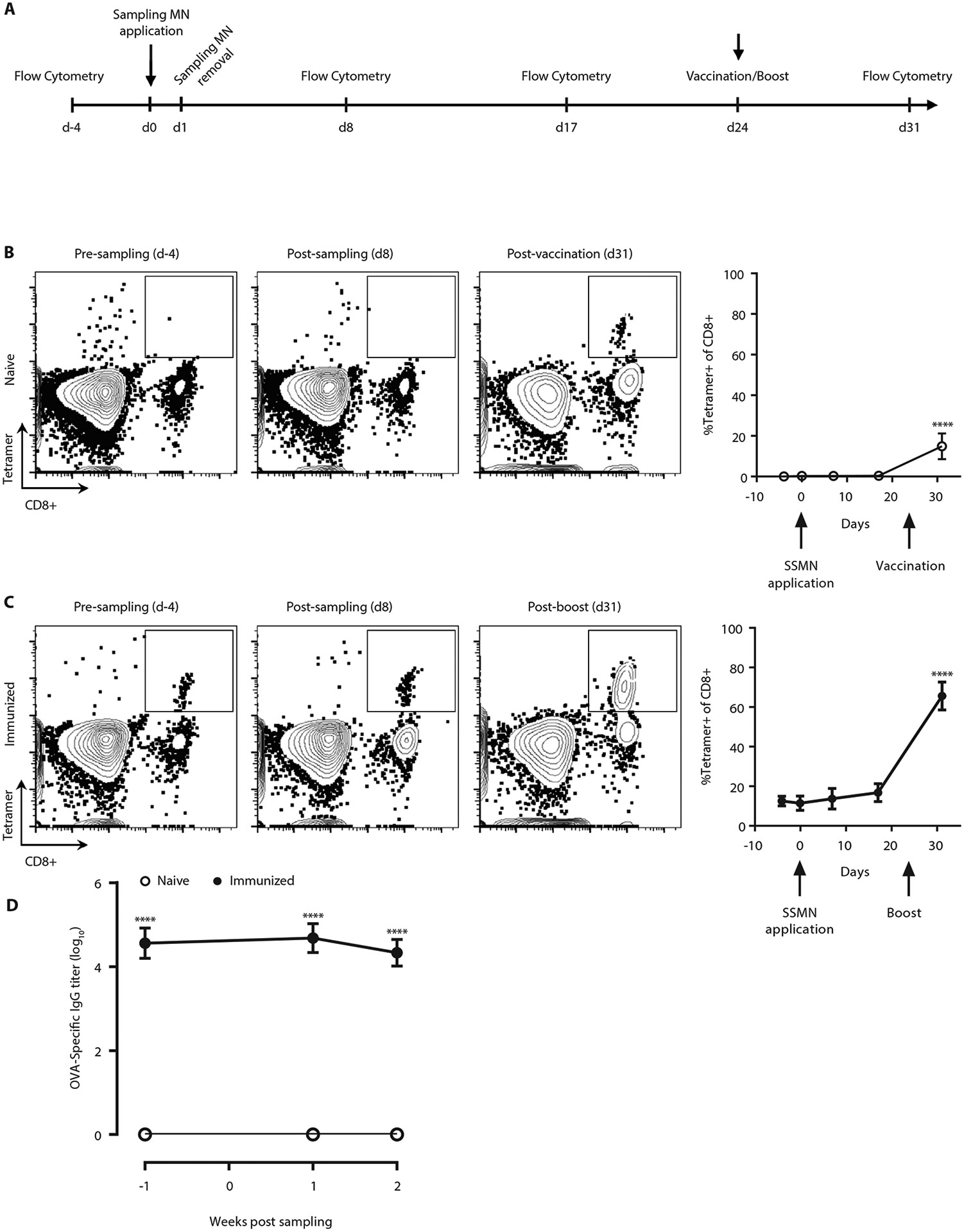
Sampling microneedles do not change the immune status of the animal. SSMN arrays containing adjuvants and ICMVs loaded with 2 μg of OVA were applied to the ears of naïve or OVA-immunized C57Bl/6 mice, immunized 8-10 weeks prior, (*n*=5/group) for 24 hrs, then retrieved and analyzed via flow cytometry. Shown are experimental timeline **(A),** representative flow cytometry plots and quantification of OVA-specific CD8^+^ T cells from blood in naïve **(B)** and previously immunized **(C)** mice, before and after SSMN application at day 0 and vaccination on day 24. **(D)** Serum OVA-specific IgG titers pre- and post-sampling with SSMNs. Data shown are mean ± s.e.m. from one representative of two independent experiments. ****: p < 0.0001 analyzed by one-way ANOVA, followed by Tukey’s HSD.

A potential concern with SSMNs is that recruited APCs might carry antigen from the microneedle matrix to draining lymph nodes, thereby immunizing the recipient and altering their immune status by the act of sampling. To test whether microneedle sampling immunizes animals, the skin of naïve mice was sampled with optimized SSMNs carrying adjuvants (polyI:C and pam3cys) and OVA-ICMVs (Fig. 6A). As shown in Fig. 6B, OVA-specific CD8^+^ cells in the blood remained undetectable before and after sampling. As a positive control, the animals were subsequently vaccinated at 24 days post-sampling with OVA in adjuvant, and 7 days later a clear tetramer^+^CD8^+^ T-cell population appeared in the blood. We also tested the more sensitive setting of animals having pre-existing memory T-cells, by sampling the skin of OVA-immunized mice. Again, no significant difference was found in the frequency of antigen-specific CD8^+^ cells in the blood before and after sampling (Fig. 6C). Extending the SSMN application time to 48 hours also did not immunize the animals, as shown in Fig. S8.

Antibody responses can often be elicited by low doses of antigen reaching lymph nodes even under conditions where cross presentation of antigen to CD8^+^ T-cells is negligible. We thus next assessed whether SSMNs induced changes in serum antibody titers against antigens carried in the microneedle gel matrix in the naïve or pre-immune settings. As shown in Fig. 6D, serum OVA-specific IgG titers showed no change before and after sampling with microneedles carrying OVA-ICMVs, whether applied to naïve or OVA-immunized animals. Thus, sampling of cells/ISF from the skin by SSMNs does not appear to immunize or alter pre-existing T- or B-cell responses against target antigens.

### Sampling microneedles allow longitudinal monitoring of resident-memory T cells

**Figure 7:**
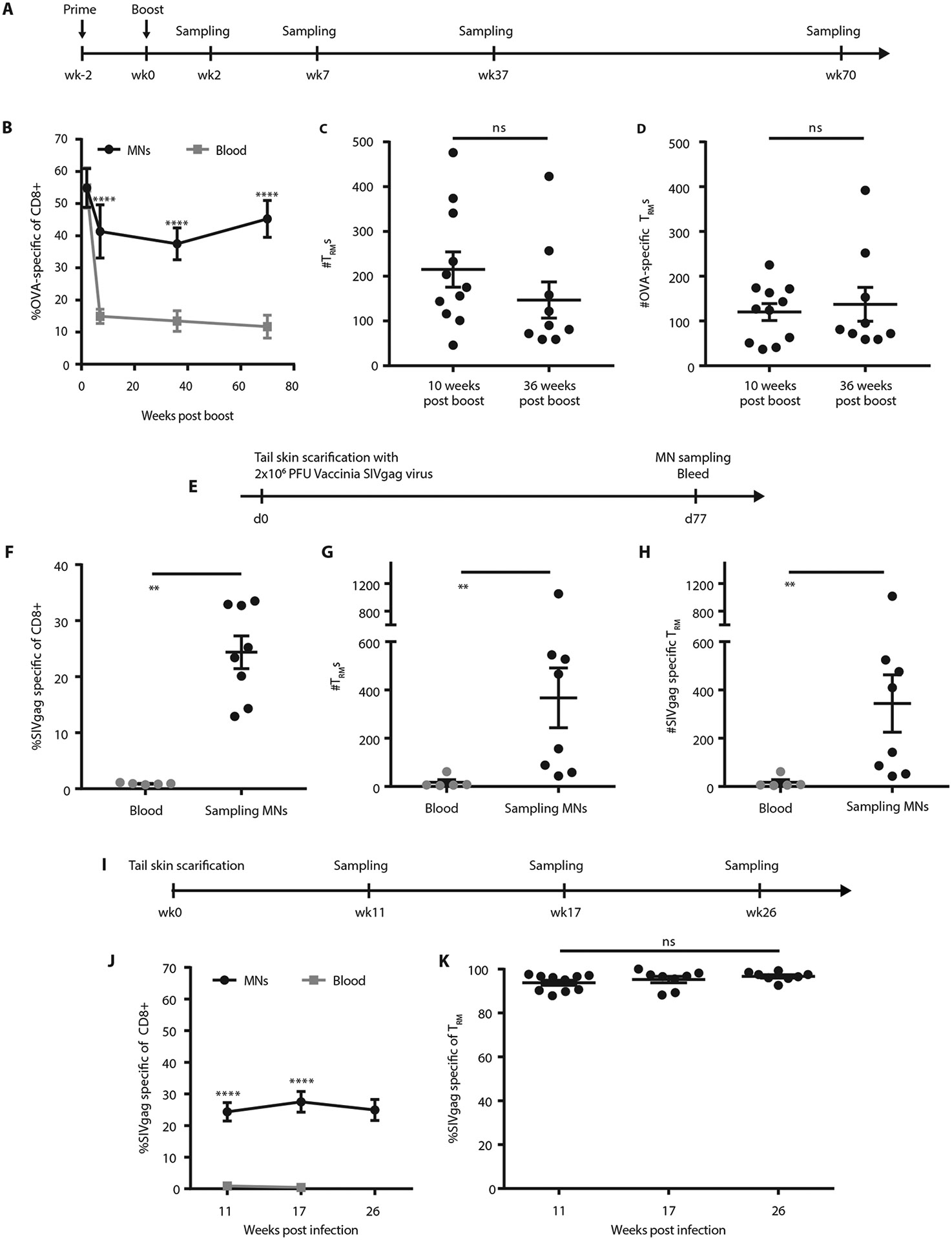
Sampling microneedles reveal a stable population of T_RM_s in skin up to a year post vaccination or infection. **(A-D)** Groups of C57Bl/6 mice (*n*=5/group) were primed and boosted with OVA and adjuvant, and then repeatedly sampled using SSMN arrays and blood draws over time. **(A)** Timeline. **(B)** OVA-specific CD8^+^ T cells over time. (**C-D**), T_RM_s (**C**) and OVA-specific T_RM_s (**D**), quantified from SSMNs and blood. **(E-H)** Groups of C57Bl/6 mice (*n*=5/group) were infected with 2x10^6^ PFU of SIVgag-expressing vaccinia virus via tail skin scarification and sampled beginning 11 weeks post infection via blood draws or SSMNs applied to the ear containing ICMVs (2 μg AL11 SIVgag peptide and 5 μg pam3Cys) and 5 μg polyI:C. After 24 hrs, patches were retrieved and analyzed via flow cytometry. Experimental timeline **(E)**, frequency of SIVgag tetramer^+^CD8^+^ cells **(F)**, enumeration of CD8^+^CD69^+^CD103^+^ T_RM_ cells **(G)** and SIVgag-specific T_RM_s **(H)** from blood and SSMNs. **(I-K)** Timeline **(I)**, frequency of SIVgag-specific CD8^+^ T cells **(J)** and frequency of SIVgag-specific T_RM_s **(K)**, when sampled at various times post vaccinia infection. Data shown are mean ± s.e.m. from one representative of 2-3 independent experiments, analyzed by Wilcox-Mann-Whitney test **(F-H)**, one-way ANOVA **(K)** or two-way ANOVA **(B, J)**, ns, nonsignificant, **p < 0.01, ***p < 0.001, ****, p < 0.0001.

Having optimized the sampling microneedle platform, we finally sought to demonstrate the capacity of this approach to follow T_RM_ populations in the skin of animals longitudinally. Mice were primed and boosted with OVA and adjuvant, then skin-resident immune populations were tracked at 2-70 weeks post-boost. As expected, the proportion of SIINFEKL tetramer^+^ (OVA-specific) CD8^+^ T-cells in blood decreased sharply in the 10 weeks following the boost, plateauing to a stable population of circulating memory cells (Fig. 7A-B). In contrast, SSMNs revealed a tissue-resident population of antigen specific T-cells that decayed slightly over the same 10-week post boost period and then remained roughly constant for the next 60 weeks. The number of T_RM_s and OVA-specific T_RM_s also remained unchanged following sampling via SSMNs (Fig. 7C-D).

We next evaluated SSMNs for measuring T_RM_s following a live infectious challenge. Mice were infected with vaccinia virus expressing SIVgag via tail skin scarification (28). SSMNs were prepared incorporating ICMV nanocapsules loaded with gag peptide, and cells were sampled from ear skin of the mice at 11 weeks post infection. The microneedle platform detected T_RM_s and gag-specific T_RM_s, which were not present in blood (Fig. 7E-H). The frequency of antigen-specific CD8^+^ cells both in the skin (sampled via microneedles) and in systemic circulation (sampled via blood) was lower than in OVA-immunized mice, as the skin-scarification model is a weaker form of antigen-exposure than intradermal immunization. However, similar to the OVA model, SSMNs detected a stable skin-resident antigen-specific T-cell population that was completely absent from the blood a few months post infection (Fig. 7I-K). Thus, SSMNs can report longitudinally on skin-resident memory cell populations not detectable in the systemic circulation.

### Immune cells can be sampled from human skin explants

**Figure 8:**
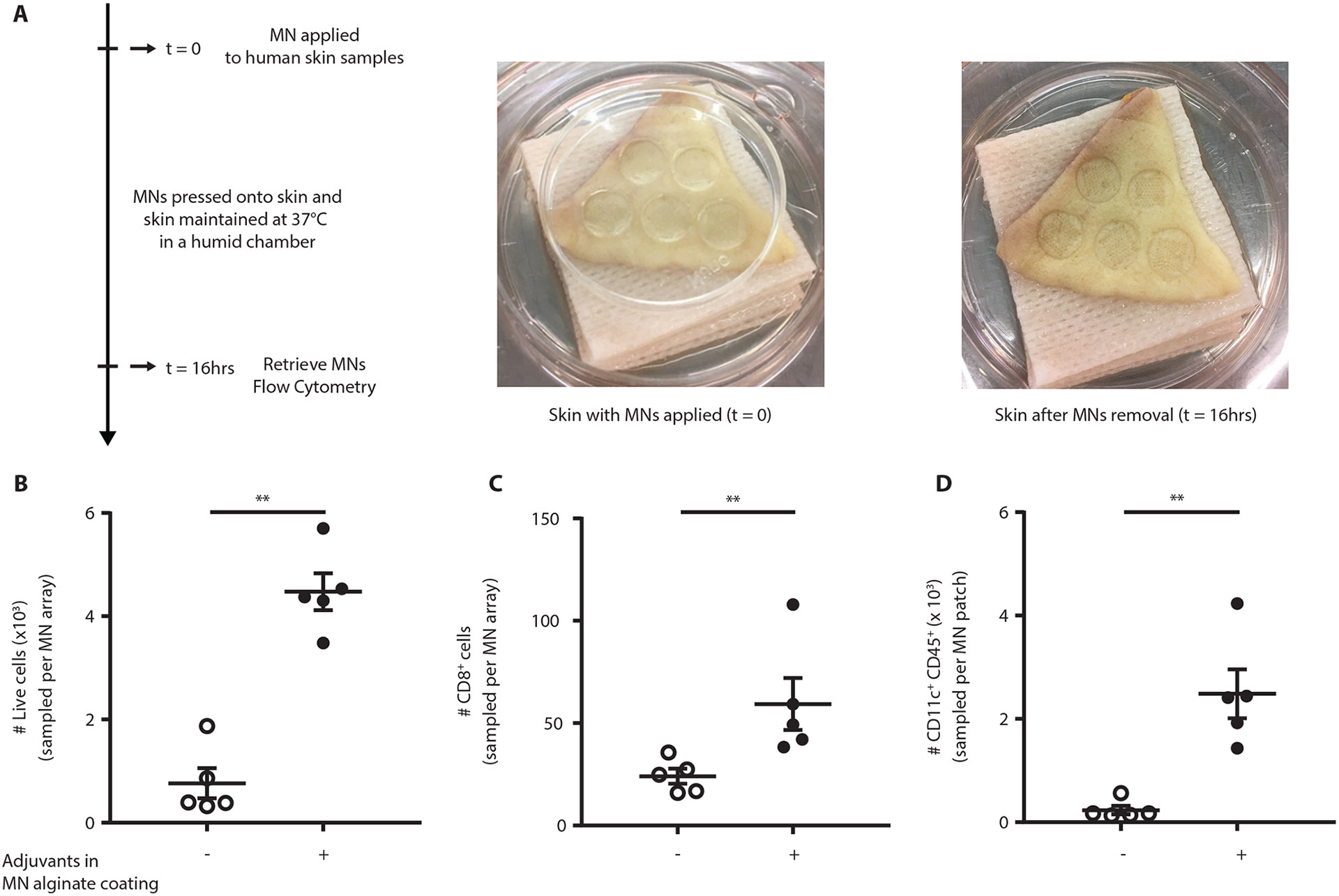
SSMNs enable sampling of lymphocytes from human skin. Five SSMN arrays each were applied to excised human skin samples from *n*=5 donors for 16 hrs, and then gel coatings were digested for analysis of recovered cells via flow cytometry. **(A)** Timeline and photographs of the experimental setup. **(B-D)** Enumeration of recovered total live cells **(B)**, CD8^+^ cells **(C)**, CD11c^+^CD45^+^ cells **(D)**, per sampling microneedle array, from microneedles containing no adjuvants, or SSMNs containing polyI:C and ICMVs encapsulating pam3Cys. Data shown are mean ± s.e.m. from two independent experiments with samples obtained from 5 total donors, analyzed by two-tailed nonparametric Mann-Whitney test, **p < 0.01.

We finally performed proof-of-concept studies to test microneedle sampling from human skin, which is thicker than mouse skin. Sampling microneedles, similar to those utilized in murine studies, with or without incorporated adjuvants were applied to fresh human skin obtained from abdominoplasty surgeries for 16 hours, maintaining the samples in a humid chamber at 37°C (Fig. 8A). Immunophenotyping via flow cytometry of sampled cells mirrored findings from the murine studies: microneedles containing adjuvants sampled several thousand live cells per patch from human skin and increased the number of CD8^+^ and CD11c^+^ cells recovered, compared to microneedles without adjuvants (Figs. 8B-D, S9). These results suggest SSMNs are suitable for minimally-invasively sampling of immune cell populations in human skin.

## Discussion

Recent studies have identified key roles for tissue-resident immune cells. T_RM_s in particular have been revealed as critical players in immunity, even in the absence of ongoing antigen presentation. T_RM_s in both mice and humans have been implicated in immune protection in the lungs, skin, gut and other mucosal linings, enhancing immunity to both infections and tumors (27). Investigation of T_RM_s and their function in small animal models is usually achieved by sacrificing cohorts at defined time points to harvest their tissue, or by obtaining invasive biopsies from larger animals and humans, since T_RM_s cannot be obtained from traditional blood draws.

Here, we present a microneedle-based, minimally invasive system for monitoring tissue-resident immune cells. This microneedle sampling platform enables isolation of live immune cells from the skin as well as ISF and biomarkers contained within it. By incorporating specific antigens within nanocapsules embedded within the cell-sampling alginate layer, SSMNs become a micro-Mantoux test and minimally-invasive biopsy in one, allowing target lymphocyte populations of interest to be enriched in the sampled population, mimicking the response to a genuine infectious challenge. Sampling microneedles take the qualitative output of a classical delayed type hypersensitivity/Mantoux test and could enable deep phenotypic and functional profiling of the responding antigen-specific tissue infiltrates. The ability to isolate live cells permits longitudinal analysis of functional traits of lymphocytes from the same animal/individual, traditional phenotyping, or advanced genomic methods such as single-cell RNA-seq. Optimized SSMNs enabled ~2,500 leukocytes to be recovered from a single 1 cm-diameter patch following a 24 hr application.

Key to this approach was the identification of a microneedle design which could be infiltrated by cells but retained sufficient mechanical integrity to withstand the mechanical forces of skin insertion. With an ultimate goal of low cost, safe, disposable patches, we focused on microneedles fabricated from melt-molded bioresorbable polymers. In preliminary studies we found that porous polymer microneedles did not have sufficient mechanical integrity for skin insertion, consistent with the rapid decline in modulus of materials as volume percent porosity increases. By utilizing a dehydrated hydrogel coating over a solid “core” microneedle substrate, we were able to arrive at a composition with a stable stiff surface layer during skin insertion, which could swell to provide a porous matrix amenable to cell infiltration *in situ*.

Sampling microneedles carrying no stimulus in the hydrogel layer could recover lymphocytes for analysis, likely attracted to the patch in response to cell death and cytokines/chemokines produced in response to the local physical micro-trauma of microneedle insertion (29). However, we incorporated specific antigens and adjuvants into the sampling layer to both increase the number of recovered cells and to more completely mimic cellular recruitment elicited by an infectious challenge- where both antigen and danger signals would naturally be present. Incorporation of adjuvants into the gel layer increased cell recovery 2-fold over “empty” alginate coatings, and introduction of specific antigens further increased this recovery another ~4.5-fold in immunized animals.

Microneedles that release antigen and adjuvant into skin tissue are well known to effectively prime immune responses *in vivo* (18, 30, 31). In response to inflammatory cues, Langerhans cells, dermal dendritic cells, and other APCs either resident or recruited to the skin will become activated, leading to upregulation of chemokine receptors that guide their migration to lymphatic vessels and draining lymph nodes, where captured antigen is presented to lymphocytes (32). We sought to circumvent this natural process during microneedle sampling by encapsulating our stimulatory antigen in nanocapsules physically entrapped within the alginate layer, to avoid dissemination of antigen into the surrounding tissue following microneedle application. SSMNs delivering small but non-negligible quantities of antigen/adjuvant did not lead to detectable priming of antigen-specific T cells or antibody responses in treated mice, even if the animals had pre-existing memory populations against the sampling antigen. The fact that patch application for up to 48 hr did not stimulate an immune response suggests that APCs recruited to the microneedle coating are unable to migrate back out of the gel layer. We hypothesize that cells recruited into the alginate layer are shielded from chemokines produced by lymphatics that normally guide APCs to the draining lymph nodes, effectively trapping them in the microneedle coating.

A limitation with collecting cell and/or ISF samples using sampling microneedles includes the size of the sample and possible limit-of-detection issues for methods that depend on high analyte concentrations. On average, ~ 5,000 live cells including ~2,500 leukocytes are obtained per microneedle array (1 cm^2^) and about 1-2 μL of ISF. Of course, increasing the size of the microneedle array will linearly increase the sample size. Additionally, with regards to safety considerations post microneedle application, similar to the findings of other studies (33), the area of sampling microneedle application in our studies also healed within 2-3 days, with only minor erythema and inflammation for the first 24-48 hours.

We expect that microneedle immune monitoring can impact at least three major areas of medicine: (1) enabling better disease management in autoimmune conditions by predicting oncoming disease flares, (2) monitoring tissue status in transplantation, and (3) monitoring vaccine responses. Methods for monitoring autoimmune diseases rely primarily on blood draws. However, cutaneous disorders such as lupus or psoriasis may be better served by biomarkers directly obtained from the skin. It is known that while ~ 83% of serum proteins are found in ISF, 50% of the proteins found in ISF are found in the interstitial fluid alone, and not in serum (21). Being able to sample for biomarkers from skin, in a painless and minimally-invasive manner, without the need of trained personnel for fluid withdrawal, could enable more granular flare-prediction. In transplantation, recent work monitoring local reactions to skin transplants has suggested that donor T_RM_s show early accumulations in allografts that will be rejected (34). A minimally-invasive means to monitor for such reactions and pre-emptively provide immunosuppressive interventions could increase the lifetime of these grafts. And finally, as shown here in our preclinical models, tissue-resident memory T cell responses established by vaccination can be readily monitored with SSMNs. We focused here on skin immune monitoring due to its importance for autoimmunity, transplants, and vaccines against bacterial and mosquito-borne pathogens, but microneedles have been employed at many other mucosal sites such as buccal (35) and vaginal (36) surfaces, as well as applied to cutaneous tumors (37). We thus expect microneedle sampling to be relevant for many other applications beyond immune monitoring in the skin.

## Materials and Methods

### Study design

The objective of this study was twofold: first, to sample resident-memory cell populations, not found in blood, from skin, via a minimally invasive skin patch, incorporating microneedles and second, to assess the quantification of commonly found biomarkers in blood, e.g. antigen-specific IgG, as detected in interstitial fluid collected by microneedles. All *in vivo* studies involving flow cytometric analysis were carried out in C57BL/6 mice, randomized into groups of four or more mice per treatment. Primary data are reported in table S1.

### Mice

Animal studies were approved by the MIT IUCAC (protocol # 0717-076-20) and animals were cared for in the USDA-inspected MIT Animal Facility under federal, state, local, and NIH guidelines for animal care. Female C57BL/6 mice 3-6 weeks of age and C57Bl/6 mice expressing GFP under control of the ubiquitin promoter were obtained from the Jackson Laboratory, and the colonies were maintained at the animal Koch Institute mouse facility at MIT.

### Fabrication of alginate-coated microneedles

PDMS molds (Sylgard 184, Dow-Corning) for fabrication of microneedle arrays were prepared by laser micromachining (38). Poly-L-lactide (Resomer L 207 S, Evonik Industries AG) was melted over the molds under vacuum (−20 mm Hg, 200°C, 40 min). Poly-L-lysine (Sigma P4832, 0.01 wt%) solution was pipetted onto PLLA microneedles for 30 mins, the solution was removed after 30 minutes, and the microneedles were dried at 25°C. Alginate (Pronova SLG 100 or Pronova SLM 20, FMC Biopolymers) and sucrose (Sigma) solution (0.35 mg alginate, 1.4 mg sucrose in 60 μL of water) was pipetted onto each PLLA microneedle array and dried under vacuum at 25°C for at least 4 hours. Crosslinking solution containing ICMV particles and poly I:C (Invivogen, HMW, average size 1.5kb – 8kb, 5 μg) and calcium (0.1 mg) in total volume of 50 μL was pipetted onto the surface of microneedles and dried under vacuum for > 12 hours.

### Skin application of microneedles

Animals were anesthetized using isofluorane and ears of the mice were laid out flat on 3M Nexcare waterproof tape. Alginate coated microneedles, (with or without cargo of antigen and/or adjuvant in the gel coating), were applied by pressing down vertically with the thumb or index finger while securing Nexcare tape around the microneedle to keep it securely in place. Another layer of waterproof tape was secured around the first layer to keep the microneedle application site dry during the application period.

### Processing of cells from gels and microneedles, and flow cytometry

Subcutaneously injected gels were mashed using the backside of a syringe plunger and digested with alginate lyase (1mg/mL, Sigma A1603) and EDTA (0.02% of a stock solution pH 5.5) for 45 mins at 37°C with intermittent pipetting to break up the gel further. The solution was strained through at 50 μm cell strainer and pelleted. Frequencies of live cells were determined following staining with DAPI. Each alginate-coated microneedle retrieved from mouse ears was immersed in 200 μL of PBS containing 1% BSA and 100 mM EDTA and incubated at 37°C on a shaker at 150 rpm for 30 mins. The supernatant was collected and centrifuged to pellet cells. Recovered cells were resuspended in 100 μL of 0.36 μM CFSE in PBS for 5 minutes at 25°C for staining, quenched with 150 μL RPMI containing 10% FBS for 15 minutes and washed. Frequencies of antigen-specific CD8^+^ T-cells and their phenotypes were determined by flow cytometry analysis of labeled cells following staining with anti-mouse antibodies (CD8α APC/Cy7, CD69 PE/Cy5, CD103 BV421) from Biolegend, and SIINFEKL/H-2K^b^ peptide–MHC tetramer (iTAg Tetramer/PE - H-2Kb OVA (SIINFEKL), from MBL) or SIVgag tetramer (iTAg Tetramer/PE - H-2Db SIV GAG (AAVKNWMTQTL) from MBL), using a BD FACS Celesta HTS-1.

### Human skin experiments

Ethics statement: Healthy human skin tissue was obtained from abdominoplastic surgery. The studies were approved by the respective institutional review boards (National Health Group Domain Specific Review Board (NHG DSRB 2012/00928) and Singhealth Centralized Institutional Review Board (CIRB 2011/327/E), respectively) and patients gave written informed consent. All skin samples were processed on the day of surgery.

Microneedle patch application to explanted skin tissue: Microneedles were applied to explanted human skin samples, and adherence was maintained using a small petri-dish to provide downward pressure. These skin samples were maintained in a humid chamber for 16 hrs at 37°C, as shown in Fig. 8A.

Flow cytometry: CellTrace CFSE (Thermo C34554), CountBright absolute counting beads (Thermo C36950), CD3 APC (UCHT1, Biolegend), CD4 PE (RPA-T4, Biolegend), CD8 AF700 RPA-T8, Biolegend), CD45 V500 (HI30, BD Horizon), HLA-DR PECy7 (L243, BD Pharmingen), CD11c V450 (B-Ly6, BD Horizon), CD14 PerCPCy5.5 (HCD14, Biolegend).

### Statistical analysis

Data sets were analyzed using two-tailed nonparametric Mann-Whitney test, one or two-way analysis of variance tests, followed by Tukey’s HSD test for multiple comparisons with Prism (GraphPad Software). p-values less than 0.05 were considered statistically significant. All values are reported as mean ± s.e.m.

## Supplementary Material

Table S1 Table S1: Primary data.

Supplemental Material Fig. S1: Optimization of alginate coating composition
Fig. S2. Analysis of fluids recovered from microneedles accurately reports on surrounding solution concentrations.
Fig. S3: T_RM_ characterization in the blood and skin compartments in OVA-immunized mice.
Fig. S4: Cell recruitment is enhanced with the inclusion of adjuvants in the alginate hydrogel coating.
Fig. S5: ICMV characterization and increased recruitment of cells into sampling microneedles when ICMVs encapsulating antigen and adjuvant are embedded in the alginate layer of sampling microneedles.
Fig. S6: The activity of polyI:C is retained upon incorporation within sampling microneedles.
Fig. S7: SSMNs containing adjuvants activate recruited APCs.
Fig. S8: SSMN application for up to 48 hours does not change the immune status of the animal.
Fig. S9: Gating strategy for cells obtained from sampling microneedles applied to human skin.

## References

[1] Parapia LA, History Of Bloodletting By Phlebotomy. Br. J. Haematol 143, 490–495 (2008). 18783398 10.1111/j.1365-2141.2008.07361.x

[2] Streitz M, Miloud T, Kapinsky M, Reed MR, Magari R, Geissler EK, Hutchinson JA, Vogt K, Schlickeiser S, Kverneland AH, Meisel C, Volk H-D, Sawitzki B, Standardization Of Whole Blood Immune Phenotype Monitoring For Clinical Trials: Panels And Methods From The One Study. Transplant. Res 2, 17 (2013). 24160259 10.1186/2047-1440-2-17 PMC3827923

[3] Srinivasan V, Pamula V, Pollack M, Fair R, Clinical Diagnostics On Human Whole Blood, Plasma, Serum, Urine, Saliva, Sweat, And Tears On A Digital Microfluidic Platform, Proc. MicroTAS 1287–1290 (2003).

[4] Gomez Perdiguero E, Geissmann F, The Development And Maintenance Of Resident Macrophages. Nat. Rev. Immunol 17, 2–8 (2016). 10.1038/ni.3341 PMC4950995 26681456

[5] He Y, Shimoda M, Ono Y, Villalobos IB, Mitra A, Konia T, Grando SA, Zone JJ, Maverakis E, Persistence Of Autoreactive IgA-Secreting B Cells Despite Multiple Immunosuppressive Medications Including Rituximab. JAMA Dermatology 151, 646–650 (2015). 25901938 10.1001/jamadermatol.2015.59

[6] Sojka DK, Plougastel-Douglas B, Yang L, Pak-Wittel MA, Artyomov MN, Ivanova Y, Zhong C, Chase JM, Rothman PB, Yu J, Riley JK, Zhu J, Tian Z, Yokoyama WM, Tissue-Resident Natural Killer (NK) Cells Are Cell Lineages Distinct From Thymic And Conventional Splenic NK Cells. Elife 3, E01659 (2014). 24714492 10.7554/eLife.01659 PMC3975579

[7] Steinert EM, Schenkel JM, Fraser KA, Beura LK, Manlove LS, Igyártó BZ, Southern PJ, Masopust D, Quantifying Memory CD8 T Cells Reveals Regionalization Of Immunosurveillance. Cell 161, 737–749 (2015). 25957682 10.1016/j.cell.2015.03.031 PMC4426972

[8] Watanabe R, Gehad A, Yang C, Scott LL, Teague JE, Schlapbach C, Elco CP, Huang V, Matos TR, Kupper TS, Clark RA, Human Skin Is Protected By Four Functionally And Phenotypically Discrete Populations of Resident And Recirculating Memory T Cells. Sci. Transl. Med 7, 279ra39 (2015). 10.1126/scitranslmed.3010302 PMC4425193 25787765

[9] Beura LK, Masopust D, Snapshot: Resident Memory T Cells. Cell 157, 1488–1488.e1 (2014). 24906159 10.1016/j.cell.2014.05.026

[10] Vukmanovic-Stejic M, Reed JR, Lacy KE, Rustin MHA, Akbar AN, Mantoux Test As A Model For A Secondary Immune Response In Humans. Immunol. Lett 107, 93–101 (2006). 16979761 10.1016/j.imlet.2006.08.002

[11] Spergel JM, Brown-Whitehorn T, The Use Of Patch Testing In The Diagnosis Of Food Allergy. Curr. Allergy Asthma Rep 5, 86–90 (2005). 15659270 10.1007/s11882-005-0061-5

[12] Spiewak R, Patch Testing For Contact Allergy And Allergic Contact Dermatitis. Open Allergy J. 1, 42–51 (2008).

[13] Tatovic D, Young P, Kochba E, Levin Y, Wong FS, Dayan CM, Fine-Needle Aspiration Biopsy Of The Lymph Node: A Novel Tool For The Monitoring Of Immune Responses After Skin Antigen Delivery. J. Immunol 195, 386–392 (2015). 26026065 10.4049/jimmunol.1500364

[14] Bjerke JR, Livden JK, Degre M, Matre R, Interferon In Suction Blister Fluid From Psoriatic Lesions. Br. J. Dermatol 108, 295–299 (1983). 6187354 10.1111/j.1365-2133.1983.tb03967.x

[15] Prausnitz MR, Engineering Microneedle Patches for Vaccination and Drug Delivery to Skin, Annu. Rev. Chem. Biomol. Eng 8, 177–200 (2017). 28375775 10.1146/annurev-chembioeng-060816-101514

[16] Wang PM, Cornwell M, Prausnitz MR, Minimally Invasive Extraction Of Dermal Interstitial Fluid For Glucose Monitoring Using Microneedles. Diabetes Technol. Ther 7, 131–141 (2005). 15738711 10.1089/dia.2005.7.131

[17] Depelsenaire ACI, Meliga SC, Mcneilly CL, Pearson FE, Coffey JW, Haigh OL, Flaim CJ, Frazer IH, Kendall MAF, Colocalization Of Cell Death With Antigen Deposition In Skin Enhances Vaccine Immunogenicity. J. Invest. Dermatol 134, 2361–2370 (2014). 24714201 10.1038/jid.2014.174 PMC4216316

[18] DeMuth PC, Garcia-Beltran WF, Ai-Ling ML, Hammond PT, Irvine DJ, Composite Dissolving Microneedles for Coordinated Control of Antigen and Adjuvant Delivery Kinetics in Transcutaneous Vaccination, Adv. Funct. Mater 23, 161–172 (2013). 23503923 10.1002/adfm.201201512 PMC3595545

[19] Hong J, Shah NJ, Drake AC, DeMuth PC, Lee JB, Chen J, Hammond PT, Graphene Multilayers as Gates for Multi-week Sequential Release of Proteins from Surfaces, ACS Nano 6, 81–8 (2012). 22176729 10.1021/nn202607r PMC4040355

[20] Moon JJ, Suh H, Bershteyn A, Stephan MT, Liu H, Huang B, Sohail M, Luo S, Um SH, Khant H, Goodwin JT, Ramos J, Chiu W, Irvine DJ, Interbilayer-crosslinked Multilamellar Vesicles as Synthetic Vaccines for Potent Humoral and Cellular Immune Responses, Nat. Mater 10, 243–51 (2011). 21336265 10.1038/nmat2960 PMC3077947

[21] Kool J, Reubsaet L, Wesseldijk F, Maravilha RT, Pinkse MW, D’Santos CS, Van Hilten JJ, Zijlstra FJ, Heck AJR, Suction Blister Fluid as Potential Body Fluid for Biomarker Proteins, Proteomics 7, 3638–3650 (2007). 17890648 10.1002/pmic.200600938

[22] Hedger MP, Herriarachchi S, Measurement of Immunoglobulin G Levels in Adult Rat Testicular Interstitial Fluid and Serum, J. Androl 15, 583–590 (1994). 7721660

[23] Weldon WC, Zarnitsyn VG, Esser ES, Taherbhai MT, Koutsonanos DG, Vassilieva EV, Skountzou I, Prausnitz MR, Compans RW, Rodrigues MM, Ed. Effect of Adjuvants on Responses to Skin Immunization by Microneedles Coated with Influenza Subunit Vaccine, PLoS One 7, e41501 (2012). 22848514 10.1371/journal.pone.0041501 PMC3405087

[24] Lai Y, Di Nardo A, Nakatsuji T, Leichtle A, Yang Y, Cogen AL, Wu Z-R, Hooper LV, Schmidt RR, von Aulock S, Radek KA, Huang C-M, Ryan AF, Gallo RL, Commensal Bacteria Regulate Toll-like Receptor 3–dependent Inflammation After Skin Injury, Nat. Med 15, 1377–1382 (2009). 19966777 10.1038/nm.2062 PMC2880863

[25] Weninger W, Biro M, Jain R, Leukocyte Migration in the Interstitial Space of Non-lymphoid Organs, Nat. Rev. Immunol 14, 232–46 (2014). 24603165 10.1038/nri3641

[26] Guermonprez P, Saveanu L, Kleijmeer M, Davoust J, van Endert P, Amigorena S, ER–phagosome Fusion Defines an MHC Class I Cross-presentation Compartment in Dendritic Cells, Nature 425, 397–402 (2003). 14508489 10.1038/nature01911

[27] Schenkel JM, Fraser KA, Beura LK, Pauken KE, Masopust D, Vezys V, Masopust D, Resident Memory CD8 T Cells Trigger Protective Innate and Adaptive Immune Responses, Science 346, 98–101 (2014). 25170049 10.1126/science.1254536 PMC4449618

[28] Liu L, Fuhlbrigge RC, Karibian K, Tian T, Kupper TS, Dynamic Programing of CD8+ T cell Trafficking After Live Viral Immunization, Immunity 25, 511–520 (2006). 16973385 10.1016/j.immuni.2006.06.019

[29] Coffey JW, Corrie SR, Kendall MAF, Early Circulating Biomarker Detection Using a Wearable Microprojection Array Skin Patch, Biomaterials 34, 9572–9583 (2013). 24044999 10.1016/j.biomaterials.2013.08.078

[30] Sullivan SP, Koutsonanos DG, Del Pilar Martin M, Lee JW, Zarnitsyn V, Choi S-O, Murthy N, Compans RW, Skountzou I, Prausnitz MR, Dissolving Polymer Microneedle Patches for Influenza Vaccination., Nat. Med 16, 915–20 (2010). 20639891 10.1038/nm.2182 PMC2917494

[31] Zaric M, Lyubomska O, Poux C, Hanna ML, McCrudden MT, Malissen B, Ingram RJ, Power UF, Scott CJ, Donnelly RF, Kissenpfennig A, Dissolving Microneedle Delivery of Nanoparticle-Encapsulated Antigen Elicits Efficient Cross-Priming and Th1 Immune Responses by Murine Langerhans Cells, J. Invest. Dermatol 135, 425–434 (2015). 25243789 10.1038/jid.2014.415

[32] Mildner A, Jung S, Development and Function of Dendritic Dell Subsets, Immunity 40, 642–656 (2014). 24837101 10.1016/j.immuni.2014.04.016

[33] Mooney K, McElnay JC, Donnelly RF, Children’s Views on Microneedle Use as an Alternative to Blood Sampling for Patient Monitoring, Int. J. Pharm. Pract 22, 335–344 (2014). 24308565 10.1111/ijpp.12081

[34] Lian CG, Bueno EM, Granter SR, Laga AC, Saavedra AP, Lin WM, Susa JS, Zhan Q, Chandraker AK, Tullius SG, Pomahac B, Murphy GF, Biomarker Evaluation of Face Transplant Rejection: Association of Donor T cells with Target Cell Injury, Mod. Pathol 27, 788–99 (2014). 24434898 10.1038/modpathol.2013.249

[35] Ma Y, Tao W, Krebs SJ, Sutton WF, Haigwood NL, Gill HS, Vaccine Delivery to the Oral Cavity Using Coated Microneedles Induces Systemic and Mucosal Immunity, Pharm. Res 31, 2393–2403 (2014). 24623480 10.1007/s11095-014-1335-1 PMC4163144

[36] Wang N, Zhen Y, Jin Y, Wang X, Li N, Jiang S, Wang T, Combining Different Types of Multifunctional Liposomes Loaded with Ammonium Bicarbonate to Fabricate Microneedle Arrays as a Vaginal Mucosal Vaccine Adjuvant-dual Delivery System (VADDS), J. Control. Release 246, 12–29 (2017). 27986552 10.1016/j.jconrel.2016.12.009

[37] Zeng Q, Gammon JM, Tostanoski LH, Chiu Y-C, Jewell CM, *In Vivo* Expansion of Melanoma-specific T Cells Using Microneedle Arrays Coated with Immune-polyelectrolyte Multilayers, ACS Biomater. Sci. Eng 3, 195–205 (2017). 28286864 10.1021/acsbiomaterials.6b00414 PMC5338335

[38] Demuth PC, Moon JJ, Suh H, Hammond PT, Irvine DJ, Releasable Layer-by-layer Assembly of Stabilized Lipid Nanocapsules on Microneedles for Enhanced Transcutaneous Vaccine Delivery, ACS Nano 6, 8041–8051 (2012). 22920601 10.1021/nn302639r PMC3475723

[39] Liu H, Moynihan KD, Zheng Y, Szeto GL, Li AV, Huang B, Van Egeren DS, Park C, Irvine DJ, Structure-based Programming of Lymph-node Targeting in Molecular Vaccines, Nature 507, 519–22 (2014) 24531764 10.1038/nature12978 PMC4069155

