# Supplementary material for: Cell- and Fluid-Sampling Microneedle Patches for Monitoring Tissue-Resident Immunity
Source: Sci Transl Med. Author manuscript; Available in PMC 2025 Apr 5. (PMC11972007; doi:10.1126/scitranslmed.aar2227)
Supplement: Supplemental Material — Fig. S1: Optimization of alginate coating composition Fig. S2. Analysis of fluids recovered from microneedles accurately reports on surrounding solution concentrations. Fig. S3: TRM characterization in the blood and skin compartments in OVA-immunized mice. Fig. S4: Cell recruitment is enhanced with the inclusion of adjuvants in the alginate hydrogel coating. Fig. S5: ICMV characterization and increased recruitment of cells into sampling microneedles when ICMVs encapsulating antigen and adjuvant are embedded in the alginate layer of sampling microneedles. Fig. S6: The activity of polyI:C is retained upon incorporation within sampling microneedles. Fig. S7: SSMNs containing adjuvants activate recruited APCs. Fig. S8: SSMN application for up to 48 hours does not change the immune status of the animal. Fig. S9: Gating strategy for cells obtained from sampling microneedles applied to human skin. [file NIHMS2066080-supplement-Supplemental_Material.docx]

**Supplementary Materials:**

Supplementary Material for

Cell- and Fluid-Sampling Microneedle Patches for Monitoring Tissue-Resident Immunity

Anasuya Mandal, Archana V. Boopathy, Lionel K.W. Lam, Kelly D. Moynihan, Mary E. Welch, Nitasha R. Bennett, Michelle E. Turvey, Nikki Thai, Jenny H. Van, J. Christopher Love, Paula T. Hammond* & Darrell J. Irvine*

*Corresponding author. E-mail: [djirvine@mit.edu](mailto:djirvine@mit.edu) and hammond@mit.edu

This file includes:

Materials and Methods

Fig. S1: Optimization of alginate coating composition

Fig. S2. Analysis of fluids recovered from microneedles accurately reports on surrounding solution concentrations.

Fig. S3: TRM characterization in the blood and skin compartments in OVA-immunized mice.

Fig. S4: Cell recruitment is enhanced with the inclusion of adjuvants in the alginate hydrogel coating.

Fig. S5: ICMV characterization and increased recruitment of cells into sampling microneedles when ICMVs encapsulating antigen and adjuvant are embedded in the alginate layer of sampling microneedles.

Fig. S6: The activity of polyI:C is retained upon incorporation within sampling microneedles.

Fig. S7: SSMNs containing adjuvants activate recruited APCs.

Fig. S8: SSMN application for up to 48 hours does not change the immune status of the animal.

Fig. S9: Gating strategy for cells obtained from sampling microneedles applied to human skin.

Table S1: Primary data.

**Materials and Methods**

*Synthesis of ICMV lipid nanocapsules*

Lipid nanocapsules were prepared as described previously with slight modifications *(20)*. All lipids were obtained from Avanti polar lipids and used without modification. Dried lipid films consisting of DOPC (1,2-dioleoyl-sn-glycero-3-phosphocholine) and MPB {1,2-dioleoyl-sn-glycero-3-phosphoethanolamine-N-[4-(pmaleimidophenyl)butyramide]} at a 1:1 molar ratio were rehydrated with cargo protein/peptide solutions (OVA protein, final concentration 0.1 mg/mL or AL11 peptide 0.1 mg/mL) and pam3Cys (Invivogen, final concentration 0.25 mg/mL) in 20mM bis-tris-propane in water (pH 7.0), followed by sonication and addition of dithiothreitol and CaCl_2_ at final concentrations of 3 and 40 mM and incubation at 37°C for 1 hour, respectively, to induce fusion and cross-linking of lipid bilayers, followed by washing and PEGylation (MPEG-SH-2000, Laysan Bio) by adding 100 μL of 20 mg/mL PEG-thiol and incubating at 37°C for 30 minutes, followed by washing to obtain nanocapsules.

*Atomic Force Microscopy measurements of alginate modulus*

100 μL each of 1 wt % or 2 wt % solutions of SLM20 or SLG100 (Pronova) in PBS were mixed with 6.25μL of 5 wt% CaCl2 in inserts of a 24-well Transwell plate. The mixture was immediately mixed via pipette after the addition of the CaCl2 solution. The plate was then incubated for 15 minutes at 37°C, and 100 μL of PBS was added to each sample and allowed to swell for 24 hours at 37°C. The bottom membrane of the Transwell chambers was cut out and samples were pushed out onto glass slides with flat surface up. Elastic moduli of the wet gels were then measured using an MFP-3D-Bio AFM (Asylum Research) at the MIT Center for Materials Science and Engineering in force mode.

*Subcutaneous gel injection studies*

6-10 week old female C57BL/6 mice or UBI-GFP mice were co-injected with separate syringes containing 100 μL of alginate solution (SLG100 or SLM120), and 5 wt% calcium chloride solution in 20 μL water, under the dorsal flank skin. Animals were euthanized after 24 hours and gels were collected upon necropsy. Gels retrieved from UBI-GFP mice were imaged by confocal microscopy or analyzed via flow cytometry.

*Immunizations*

Lipid-conjugated CpG, a TLR9 agonist, was used as adjuvant for model protein immunizations. The sequence used was murine ODN class B sequence 1826 with two guanine spacers: 5’-*G*G*T*C*C*A*T*G*A*C*G*T*T*C*C*T*G*A*C*G*T*T-3’ (*indicates phosphorothioate linkage). Solid phase DNA synthesis and 5' lipid conjugation were carried out as previously described using an ABI 394 synthesizer *(39)*. Following cleavage and deprotection, oligos were purified via RP-HPLC and quantified using UV-VIS. 6-10 week old female C57BL/6 mice were immunized via subcutaneous injection (at the tail base) with antigen (10 µg OVA, Worthington) along with 1.29 nmol lipo-CpG in 10 μL PBS. Control groups included naïve mice. Immune responses were characterized by flow cytometric analysis of antigen-specific T cell frequency (H-2K^b^/SIINFEKL peptide-MHC tetramer staining), phenotypic marker expression, and ELISA analysis were carried to determine memory vs effector state of immune response.

*Skin scarification*

6-10 week old C57BL/6 mice were anesthetized using isoflurane. Saline (5 μL) containing 2 x 10^6^ PFU Vaccinia-SIVgag virus was applied to the tail skin 1 cm from the base of the tail. The skin area was then gently scratched 25 times with a 28 ½ G needle.

*Multi-spectral Imaging Cytometry (MuSIC)*

Cells were stained as described above with a cocktail of anti-mouse antibodies (CD8α PE/Cy7, CD19 BV711, MHCII PerCP710, CD11b BV655, CD11c PE/Cy5, CD3e PerCP, CD45 BV605, Sytox Green and CD103 BV421) from Biolegend. Stained cells were loaded onto a nanowell array, with number of cells loaded per well governed by a rough Poisson distribution. The loaded nanowell arrays were then imaged with an epifluorescence microscope (Zeiss) with filter wheels at the relevant excitation and emission wavelengths. Compensation was performed with agarose beads (Agarose Beads Technologies) in 100 mM sodium carbonate. Each antibody was incubated with a separate aliquot of beads and imaged in all the fluorescent channels of interest. The percentage of spillover from other channels into a given channel was calculated from the fluorescence data and then used to compensate the acquired cell fluorescence data. Cells were identified from the obtained fluorescent images with Enumerator, an in-house image analysis software (Love Lab, MIT, love-lab.mit.edu). For each nanowell array, a spreadsheet containing cell information including nanowell IDs, fluorescent intensities in each channel, and cell sizes, was generated. This data was then further analyzed and gated using Matchbox, an in-house MATLAB-based single-cell analysis software (Love Lab, MIT).


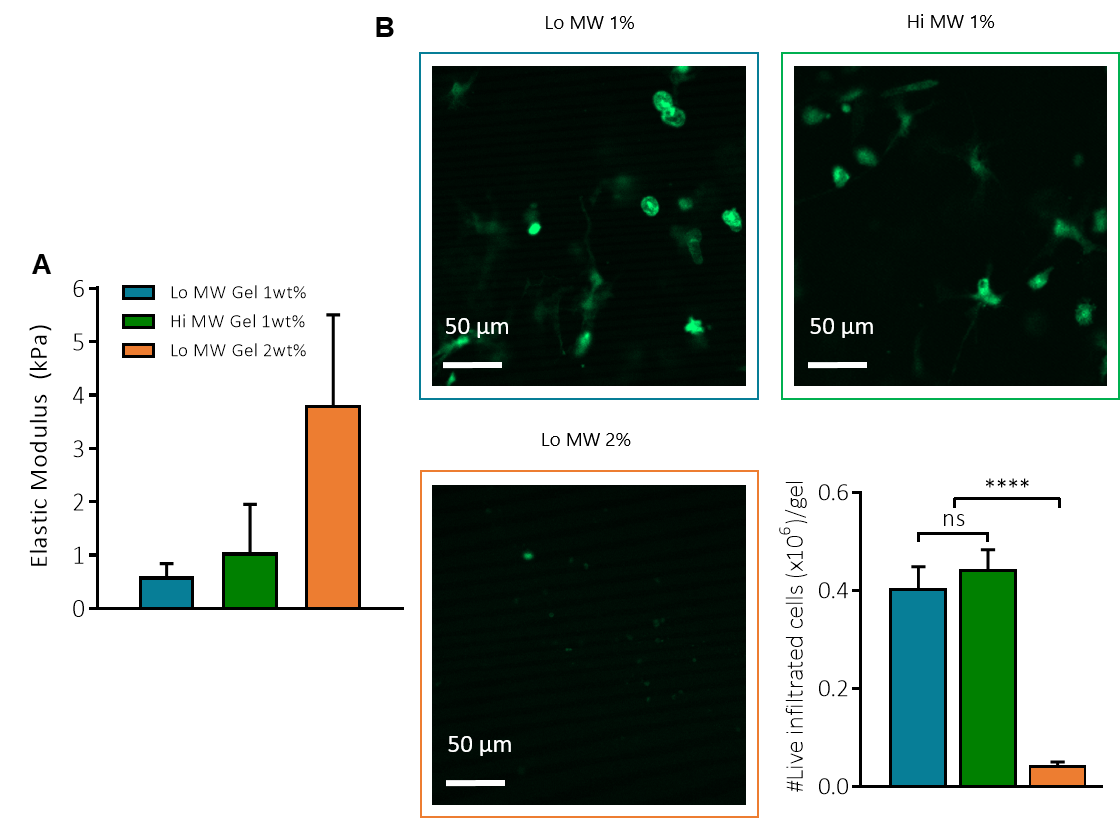


**Figure S1: Optimization of alginate coating composition.** **(A)** Elastic modulus as measured by atomic force microscopy of low (Lo, 75,000 g/mol) and high (Hi, 200,000 g/mol) molecular weight gels, at 1% and 2% wt% of gel. **(B)** Basal infiltration of cells into alginate gels when subcutaneously injected for 24 hours under the dorsal flank skin of EFGP-transgenic mice (n=5 per group), as visualized by confocal microscopy and infiltrated live cell counts using a hemocytometer, from one representative of three independent replicates. Data shown are mean ± s.e.m., analyzed by one-way ANOVA, ns, nonsignificant, ****, p < 0.0001.


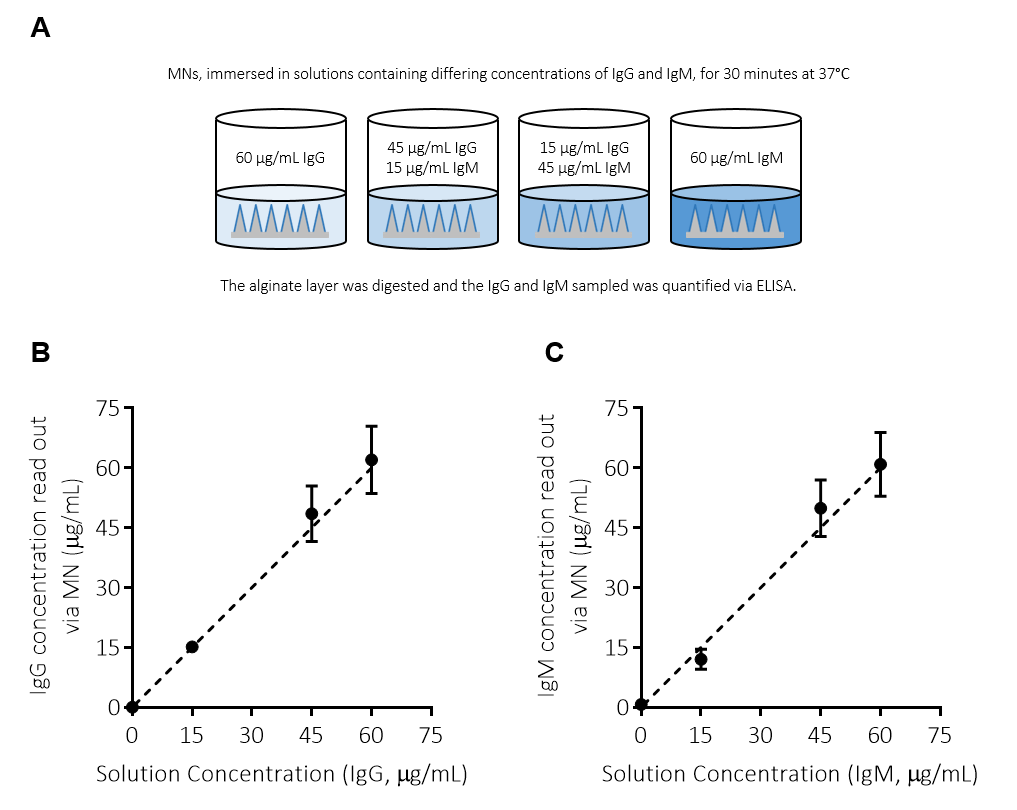


**Figure S2. Analysis of fluids recovered from microneedles accurately reflects surrounding solution concentrations. (A)** Schematic of experiment to test the accuracy of protein quantification via sampling microneedles. Briefly, sampling microneedles were immersed in solutions containing differing concentrations of IgG and IgM as model proteins. The alginate layer was then digested and the resulting solution was quantified via ELISA for IgG **(B)** and IgM **(C)**.

**
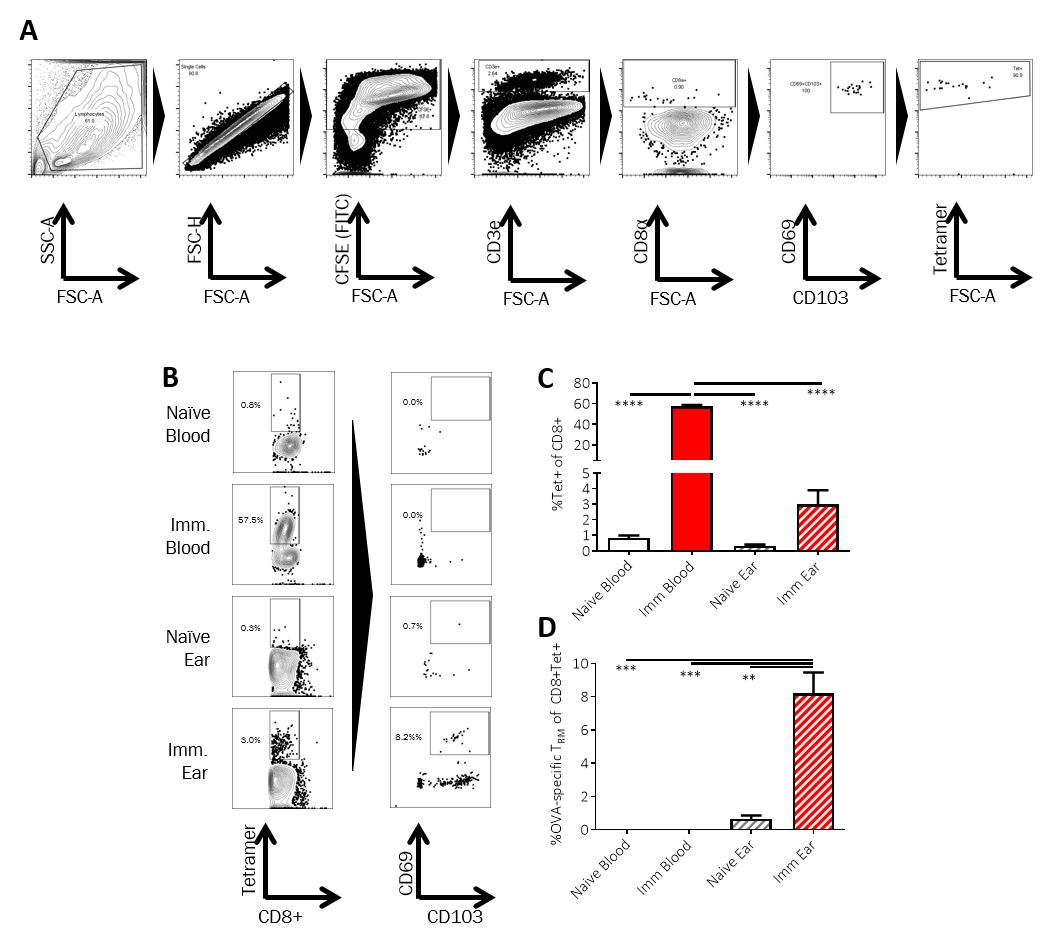
Figure S3: T_RM_ characterization in the blood and skin compartments in OVA-immunized mice.** Groups of OVA-immunized C57Bl/6 mice (*n*=3/group) were bled, euthanized, and their ears were digested and analyzed via flow cytometry. **(A)** Illustrative flow cytometric plots showing gating strategy for characterizing T_RM_s, CD3^+^C8^+^CD69^+^CD103^+^SIINFEKL^+^, in blood and digested ear tissue from mice that are naïve (“Naïve Blood”, “Naïve Ear”) or were immunized (“Imm. Blood”, “Imm. Ear”) with OVA. **(B-C)** Representative flow cytometry plots **(B)** and enumeration from groups of mice **(C)** of frequency of OVA tetramer (SIINFEKL/H-2K^b^)^+^ cells among CD8^+^ cells. **(D)** Frequency of OVA-specific CD8^+^ cells that are T_RM_ (CD69^+^CD103^+^). Data shown are mean ± s.e.m. from one representative experiment of three independent experiments. ****, p < 0.0001, analyzed by one-way ANOVA, followed by Tukey’s HSD.

**
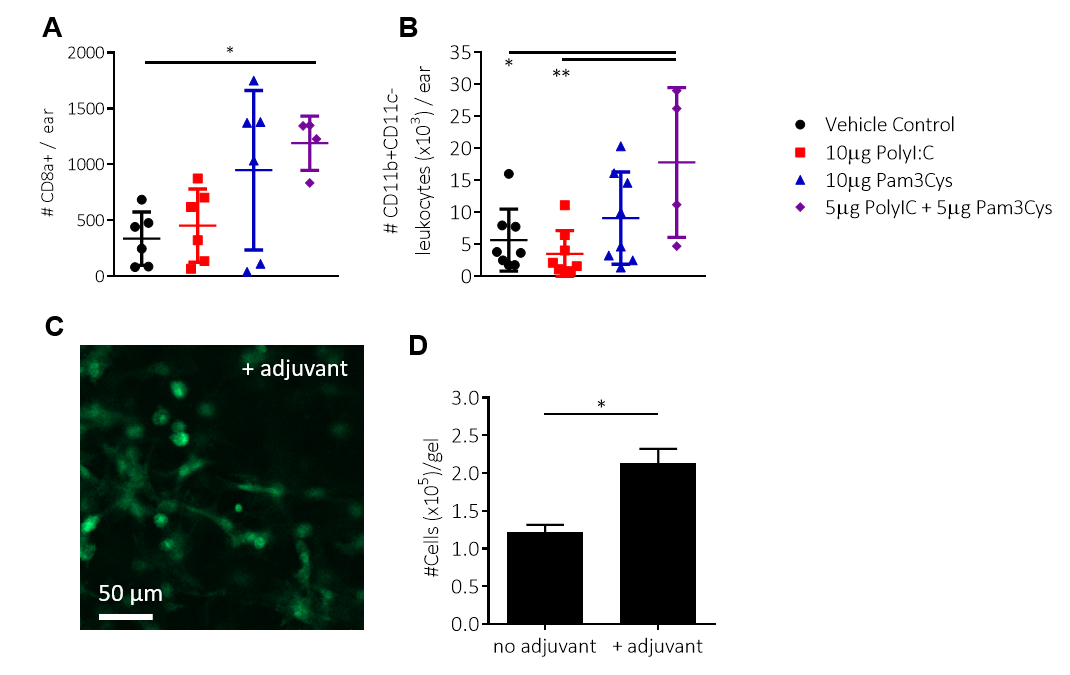
**

**Figure S4: Cell recruitment is enhanced with the inclusion of adjuvants in the alginate hydrogel coating. (A and B)** Groups of C57Bl/6 mice (*n*=6/group) were injected intradermally in their ears with adjuvants. Mice were euthanized 48 hours later and ears were digested and analyzed using flow cytometry for infiltrating populations including CD11b^+^CD11c^-^ myeloid cells **(A)** and CD8^+^ lymphocytes **(B)**. **(C, D)** Hi MW 1% alginate gels carrying 5 μg polyI:C and 5 µg pam3Cys were injected s.c. under the dorsal flank of EGFP-transgenic mice, and 24 hrs later, recovered for confocal microscopy and cell counting. **(C)** Confocal microscopy images of GFP^+^ cells infiltrating gels. **(D)** Enumeration of live cells recruited into subcutaneously injected gels under the dorsal flank of naïve C57BL6 mice for 24 hours with and without presence of adjuvants pam3Cys and polyI:C. Data shown are mean ± s.e.m. from one representative experiment of three independent experiments, *, p < 0.05 and **, p < 0.01, analyzed by one-way ANOVA, followed by Tukey’s HSD.

**
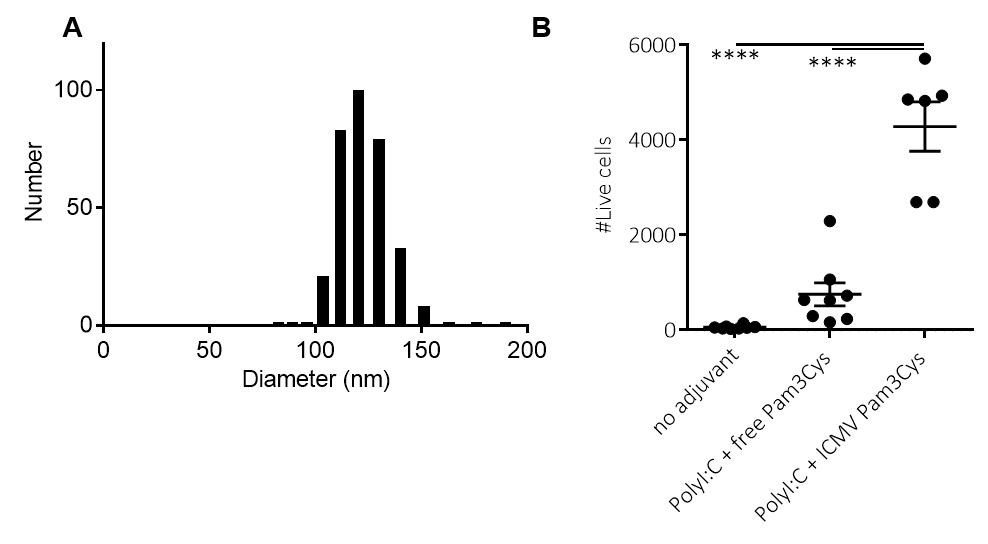
Figure S5:** **ICMV characterization and increased recruitment of cells into sampling microneedles when ICMVs encapsulating antigen and adjuvant are embedded in the alginate layer of sampling microneedles. (A)** Characterization of OVA and pam3Cys-loaded ICMVs via dynamic light scattering. **(B)** Sampling microneedles containing no adjuvant or loaded with 5 µg polyI:C and ICMVs carrying 5 µg pam3Cys in the alginate layer were applied for 12 hr to the ear skin of naïve C57BL/6 mice (*n*=8/group). Shown is enumeration of total live cells retrieved, per microneedle array. Data shown are mean ± s.e.m. from one representative experiment of two independent experiments, ****, p < 0.0001, analyzed by one-way ANOVA, followed by Tukey’s HSD.


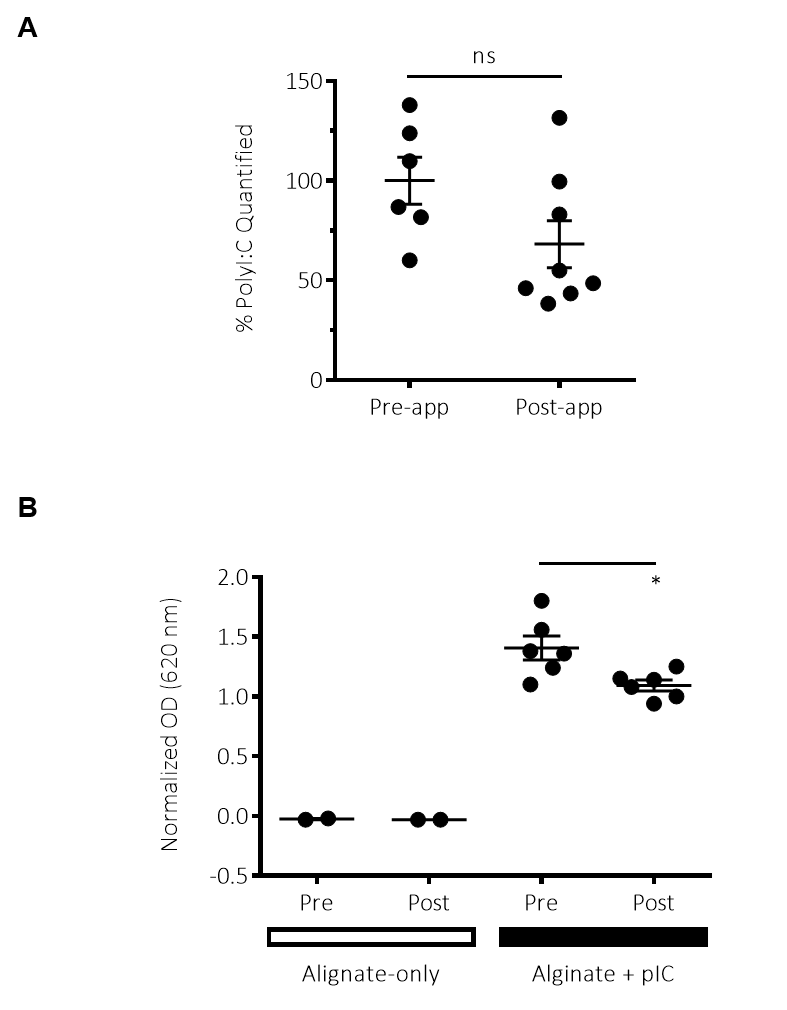


**Figure S6: The activity of polyI:C is retained upon incorporation within sampling microneedles. (A)** Microneedles loaded with rhodamine-labeled polyI:C were quantified via rhodamine fluorescence pre and post 24 hour application to mouse ears. **(B)** TLR3-expressing HEK-Blue cells were stimulated with digests from microneedles containing alginate only or alginate loaded with polyI:C, and quantified via a colorimetric assay for activation. Data shown are mean ± s.e.m. from one representative of two independent experiments, ns, nonsignificant, *, p < 0.05, analyzed by one-way ANOVA, followed by Tukey’s HSD.


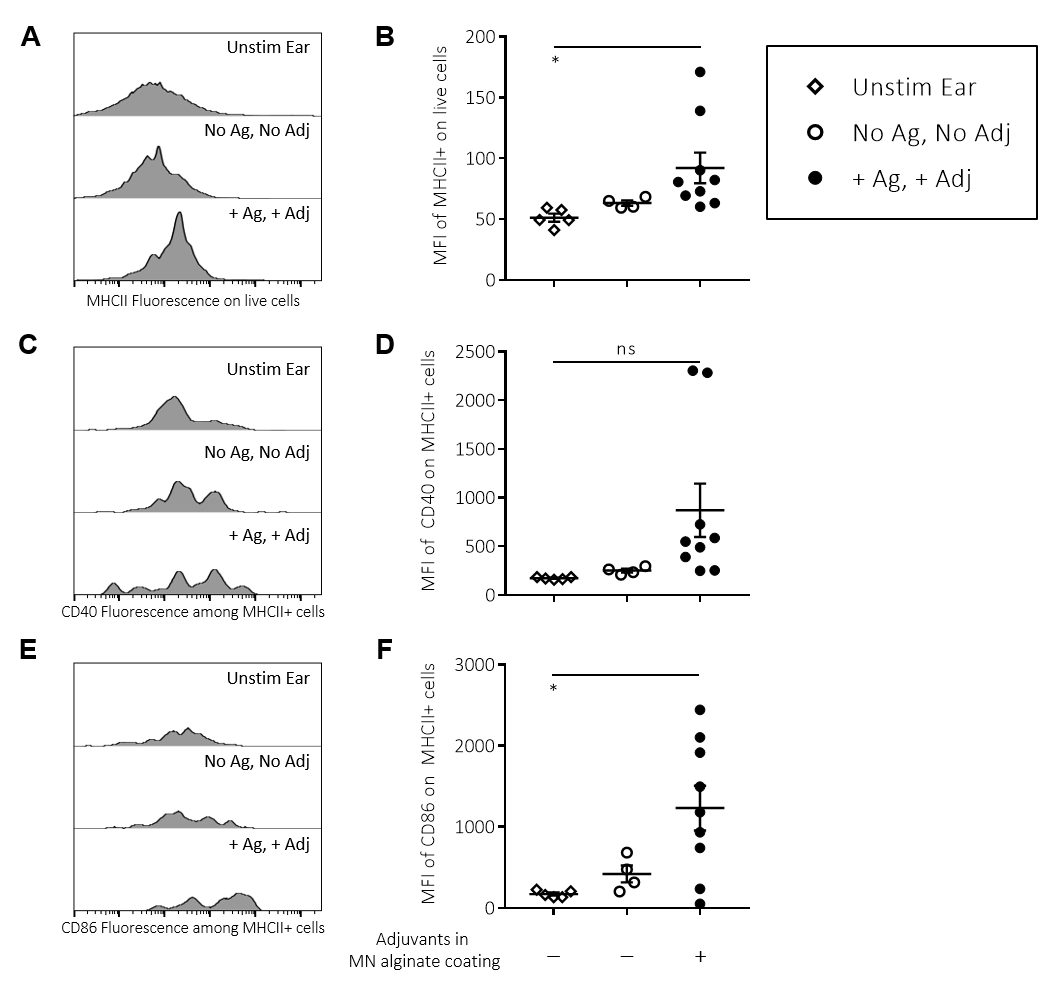


**Figure S7: SSMNs containing adjuvants activate recruited APCs.** Groups of 10 wk old naïve C57BL/6 mice were either left untreated (open diamonds), or treated for 24 hours with microneedles containing either no antigen or adjuvant (open circles) or SSMNs (solid circles). The naïve ear skin and cells sampled via microneedles were characterized by flow cytometry. **(A)** Histograms of MHCII fluorescence among live cells, **(B)** median fluorescent intensity (MFI) of MHCII on live cells, **(C)** histograms of CD40 fluorescence on MHCII^+^ cells, **(D)** MFI of CD40 on MHCII^+^ cells, **(E)** histograms and CD86 fluorescence on MHCII^+^ cells, and **(F)** MFI of CD86 on MHCII^+^ cells. Data shown are mean ± s.e.m. from one representative of two to three independent experiments. ns, nonsignificant, *, p<0.05, analyzed by one-way ANOVA, followed by Tukey’s HSD.


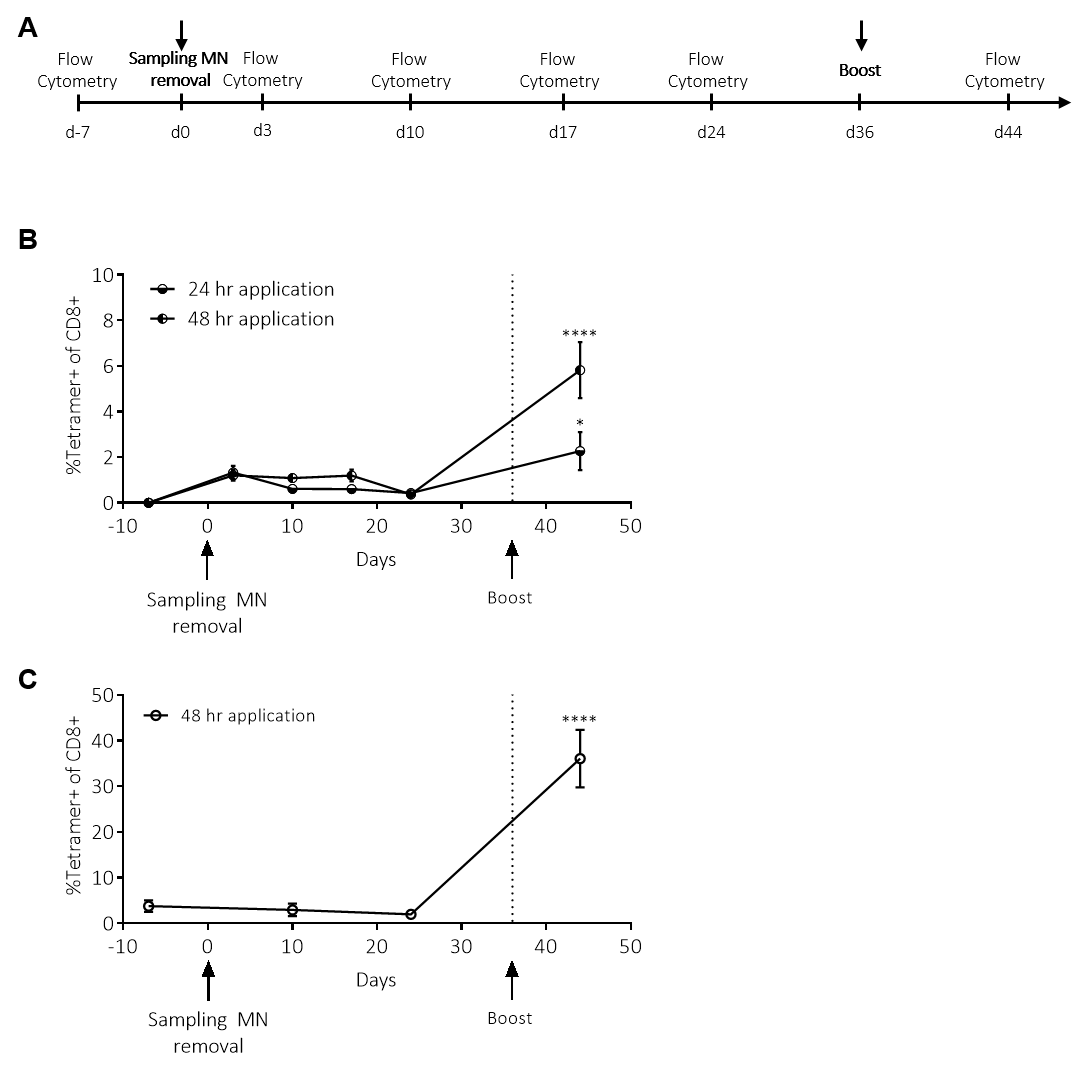


**Figure S8: SSMN application for up to 48 hours does not change the immune status of the animal.** SSMNs containing adjuvants and ICMVs loaded with 2 μg of OVA were applied to the ear skin of naïve or OVA-immunized C57Bl/6 mice (*n*=5/group) for 24 hours or 48 hours, then retrieved and analyzed via flow cytometry. Shown are the experimental timeline **(A),** quantification of OVA-specific CD8^+^ T cells from blood in naïve **(B)** and previously immunized **(C)** mice, before and after SSMN application at day 0 and vaccination on day 36. Data shown are mean ± s.e.m. from one representative of two independent experiments. ****: p < 0.0001 analyzed by one-way ANOVA, followed by Tukey’s HSD.


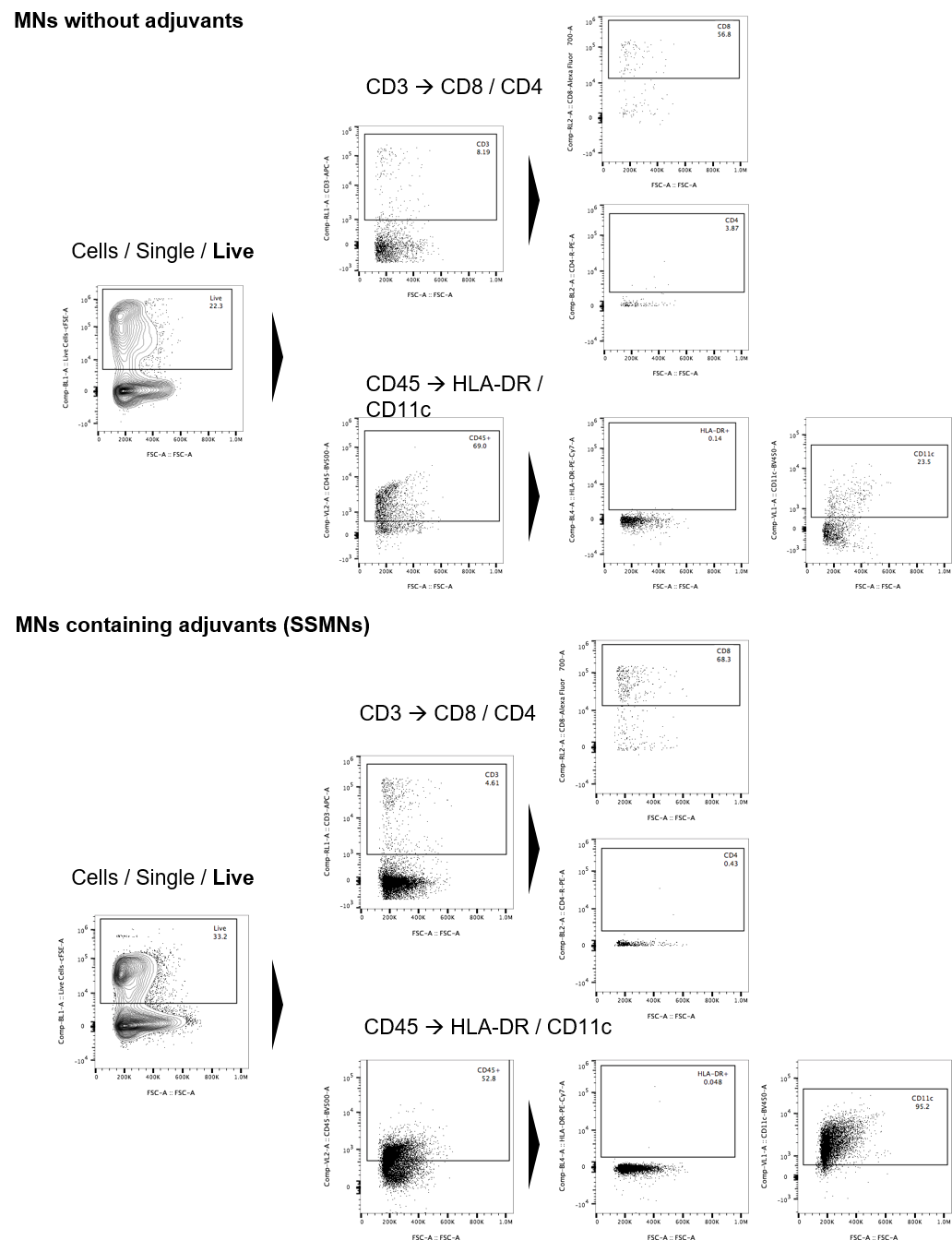


**Figure S9: Gating strategy for cells obtained from sampling microneedles applied to human skin.** Sampling microneedles, with and without adjuvants, were applied to excised human skin, and the cells sampled via microneedles were analyzed via flow cytometry. Shown here are sample flow cytometric plots showing gating strategy for characterizing CD8^+^ and CD11c^+^ cells from human skin via sampling microneedles.
